# Identification of pollen taxa by different microscopy techniques

**DOI:** 10.1371/journal.pone.0256808

**Published:** 2021-09-01

**Authors:** Matej Pospiech, Zdeňka Javůrková, Pavel Hrabec, Pavel Štarha, Simona Ljasovská, Josef Bednář, Bohuslava Tremlová

**Affiliations:** 1 Faculty of Veterinary Hygiene and Ecology, Department of Plant Origin Food Sciences, University of Veterinary Sciences Brno, Brno, Czech Republic; 2 Faculty of Mechanical Engineering, Department of Statistics and Optimization, Brno University of Technology, Brno, Czech Republic; 3 Faculty of Mechanical Engineering, Department of Computer Graphics and Geometry, Brno University of Technology, Brno, Czech Republic; University of Messina, ITALY

## Abstract

Melissopalynology is an important analytical method to identify botanical origin of honey. Pollen grain recognition is commonly performed by visual inspection by a trained person. An alternative method for visual inspection is automated pollen analysis based on the image analysis technique. Image analysis transfers visual information to mathematical descriptions. In this work, the suitability of three microscopic techniques for automatic analysis of pollen grains was studied. 2D and 3D morphological characteristics, textural and colour features, and extended depth of focus characteristics were used for the pollen discrimination. In this study, 7 botanical taxa and a total of 2482 pollen grains were evaluated. The highest correct classification rate of 93.05% was achieved using the phase contrast microscopy, followed by the dark field microscopy reaching 91.02%, and finally by the light field microscopy reaching 88.88%. The most significant discriminant characteristics were morphological (2D and 3D) and colour characteristics. Our results confirm the potential of using automatic pollen analysis to discriminate pollen taxa in honey. This work provides the basis for further research where the taxa dataset will be increased, and new descriptors will be studied.

## Introduction

Pollen is a natural component of honey, but due to its specific shape characteristics, it is successfully used as a method to identify the geographical and botanical origin of honey, or to determine a single-species honey. This issue is studied by the scientific branch called mellisopalynology. Honey analysis is based on visual inspection according to international standards of the International Honey Committee and this technique was described in detail in the work by Von Der Ohe et al. [1]. However, this method of evaluation is prone to be affected by subjective errors in analyses [1,2].

A number of different microscopic techniques are used for pollen identification. Morphological characteristics [3], especially the shape characteristics (length, width, circularity, and shape factor or length/width ratio) [4,5], are used most commonly. In light microscopy (LM) the morphological (pollen shape) and surface structures (exina) are used for the identification as well [6]. Other microscopic techniques include scanning electron microscopy (SEM) which allows a more accurate determination of the type of pollen grain based on differences in surface structures [7,8]. Transmission electron microscopy can provide information on the morphology of internal structures, but with regard to the complexity of processing, it is used mainly for descriptive studies in the field of palynology [9]. On the other hand, light microscopy is able to recognize far more taxa on species level than scanning electron microscopy [8]. Highlighting of surface structures is possible using other microscopic techniques of light microscopy, such as phase contrast (Ph), Nomarski phase, and polarization [8].

All microscopic techniques have some advantages and disadvantages. In combination they can provide complementary information about the observed objects. The BF is preferably for observations of transparent light-absorbing specimens showing a medium optical density. High-density or opaque specimens are not passed through by the transmitted light, thus fine details situated inside them or localized at the specimen’s surface cannot be well perceived. DF is preferably for observation of low-density specimens. Phase boundaries are highly contrasted by light diffraction. Lateral resolution of DF is maximized, because the incoming circular light runs to the specimen in an oblique direction[10]. DF is also able to visualize very small structures that are beyond the resolution limit, where visible diffraction patterns are generated [11]. Ph is used for visualisation of transparent specimens in light microscopy while the peripheral zones of such specimens appear in lower brightness and the central parts are highlighted up to the brightness of the surrounding medium [12,13].

Identification of the pollen taxon is based on pollen grain morphology, and the accuracy of identification may vary according to the microscopic technique used. Ullah et al. [14] used light microscopy and SEM to study pollen micro-morphology of 18 species belonging to seven genera of *Alsinoideae* subfamily. And their results showed that pollen grains of this subfamily have the common feature of pantoporate ornamentation. Another study applied scanning electron microscopy to observe and describe the ultrastructure of the exine ornamentation and aperture structure of the *Lamioideae* taxa [15]. Also the study [16] confirmed palyno-anatomical features in monocot taxa, where the correct identification of the 24 monocot taxa used the light and scanning electron microscopy.

With the increase of computer power, it is possible to use image analysis process for pollen identification. Image analysis transfers image content into feature description, such as colour, shape, and texture [17]. The colour of the pollen grain is represented by the mean values of the RGB components (Red, Green, Blue) or the hue value which, in contrast to RGB, is independent of brightness. The shape is described by geometric parameters such as shape factor, circularity, and dispersion which, unlike the area, are independent of the size of the object. The texture is generally calculated as spatial variation of the brightness intensity of the pixels [18]. But also contour–inner segmentation can be used as textural description [19]. Some authors developed different texture description. The use of image analysis for the identification and classification of pollen grains has been validated by a number of authors in various modifications [6,20–23]. Another study confirmed slight differences in the number of pollen grains in the case of human visual inspection or an automatic analysis [24]. However, only a few studies have been devoted to the identification of pollen grains in honey.

In these studies, bright field microscopy (BF) was used to recognize pollen grains. This microscopic technique is also used by the harmonized method [1]. The dark field microscopy (DF) [23], fluorescence technique [25], was validated for pollen analysis in palynology. In the study by Jacinto-Pimienta et al. [26], melissopalynology was performed using the phase contrast microscopic technique (Ph). No studies have yet been performed on the analysis of pollen in honey by other microscopic techniques using image analysis.

For the comparison of microscopic techniques, a quantitative melissopalynological technique was newly applied, this technique uses the filtration of pollen grains on a microcellulose filter, which is one of the harmonized analyses of honey according to the International Honey Committee [1]. The aim of this study is to compare three microscopic techniques (BF, DF and Ph) and their applicability for the classification of pollen grains by image analysis and application of new EDF (Extended depth of focus) and Volume descriptors for pollen classification.

## Materials and methods

Four types of honey originating from the Czech Republic were obtained in 2019 from various honey collections. These included spring blossom honey (SBH), acacia honey (AH), lime tree honey (LTH), and honeydew honey (HH). Dominant taxa in SBH were *Brassica napus (Brassica)*, *Salix* and *Pyrus/prunus*. Dominant taxa in AH were *Brassica*, *Salix*, *Pyrus/prunus*, *Phacelia campanularia* (*Phacelia*), *Robinia pseudoacacia* (*Robinia*). In LTH, dominant taxa were *Brassica*, *Salix Pyrus/prunus*, *Phacelia*, *Tilia*. HH contained *Brassica*, *Salix*, *and Asteraceae* pollen taxa. The identification of pollen taxa was performed using pollen atlases, scanning electron microscopy and confirmed by an independent examination (Intertek Group plc, GER).

Honey samples were processed for the quantitative pollen analysis in compliance with the harmonized method [1]. A 25 mm/3 μm Millipore 3 μm filter (Merck KGaA, GER) was used in a vacuum filtration assembly (ThermoFisher, USA). After filtration, the filter was dried at 40°C for 12 hours and then mounted with solacryl (Merci, CZE).

The samples were analysed under the Eclipse Ci-L microscope (Nikon, JPN) with motorized stage of Proscan III (Prior, USA). Images were captured by the DFK 23U274 camera (Imaging Source, GER). Three microscopic techniques were used with 400x magnification. The used method was bright field light microscopy, dark field microscopy, and phase contrast microscopic technique. For dark field dry condenser with a Planchromat 40x lens was used for DF; C-C phase Contrast condenser and CFI Plan Achromat DL 40X lens, N.A. 0.65, W.D. 0.56 mm, Ph2 was used for Ph. Since it is not a fully automated system, a USB4000-UV-VIS-ES spectrophotometer (Ocean Optics Inc., USA) was used to set the same lighting conditions. An identical profile of light passing through the slide in the range of 420–750 nm in a place with no pollen grains or other impurities was ensured for each measurement. The pollen grains were captured in 5 different focal planes with distances of 8 μm from each other and an extended depth of focus image (EDF) was created to be used for image analysis.

The complete procedure for image acquisition comprised several steps. After solacryl mounting, microscopic slides were scanned in random positions in order to capture 1000 pollen grains for each microscopic technique. The selection of monitored botanical taxa followed from previous studies and represented taxa typically contained in Czech honey [27,28].

**Fig 1:**
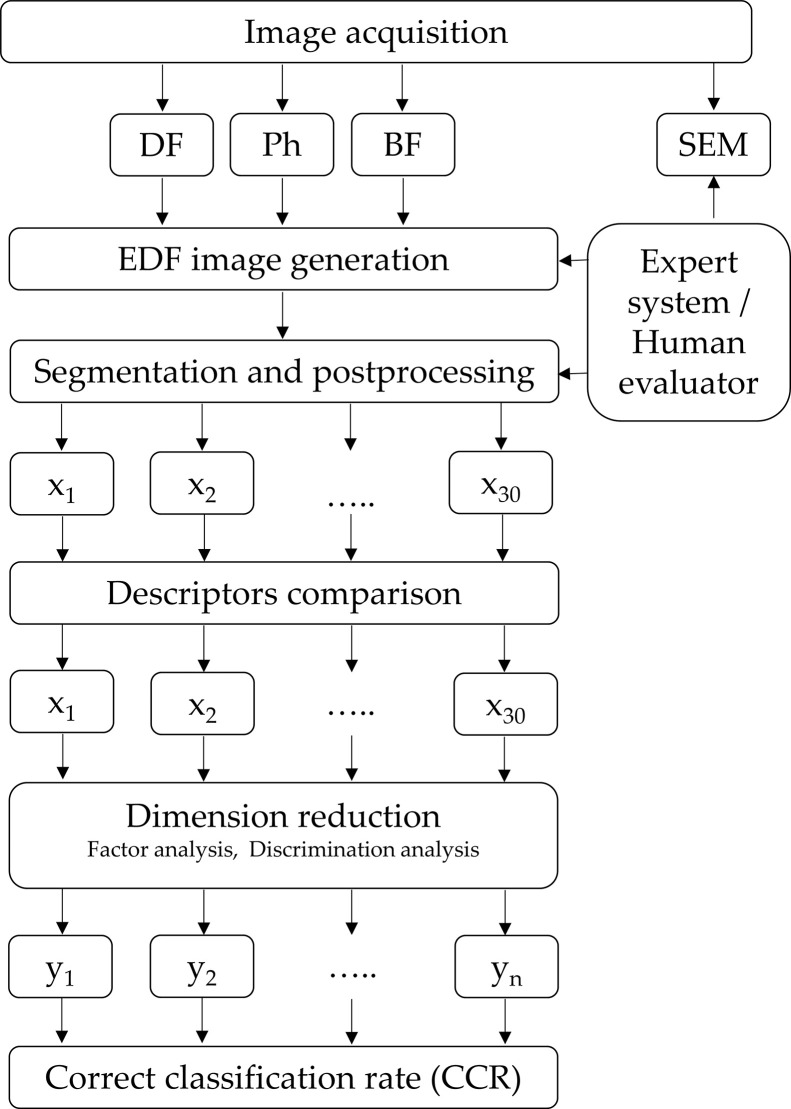
Analysis and classification methodology scheme for pollen identification.

For taxa identity confirmation, the samples were examined by SEM. After filtration, the sample was dried in a desiccator for 24 hours and applied to an aluminium double-sided adhesive tape and followed by nanocoating with a 10 nm layer of gold using the sputter coater device Q150R ES (Quorum Technologies, UK). Scanning was performed using MIRA3 (Tescan, a.s., CZE). Pollen grains was scanned in different magnifications from 4 kx to 40 kx in high vacuum. Acceleration voltage 5.0 keV and secondary electron detector were used for picture acquisition. The procedure for processing the images and the evaluation of the data obtained by image analysis is described in Fig 1.

Image analysis was performed using NIS-Elements AR 5.20 (Laboratory Imaging, CZE). Binary mask was created semi-automatically. The binarization required manual outline corrections. But a higher level of robustness, which is expected in dual segmentation, is better for our comparative hypothesis [19].

Thresholding method to the RGB colour space was applied, the thresholding range is indicated in Table 1. Object smoothing filter, and object cleaning filter, opening algorithms were used as postprocessing operation. Object cleaning filter removed small objects from the image. Opening algorithms are based on erosion followed by dilation to smooth object. Object smoothing filter smooths the binary image contours. Similar thresholding methods were used by other authors [19,29].

**Table 1:** Processing setting of objects analysis for microscopic techniques used.

	BF	DF	Ph
Thresholding binarization on RGB colour space in range	Define Threshold (85,80,62,130,122,98)*	Define Threshold (100,107,81,169,154,118)*	Define Threshold (71,72,59,136,132,90)*
Object smoothing filter (Size)		16	
Object cleaning filter (Object size)		16	16
Opening algorithms (Kernel size)		6 times 3x3	3 times 3x3

*Define pixel threshold (Red Low, Green Low, Blue Low, Red high, Green High, Blue High).

The measured morphological descriptors included 2D descriptors of Area, Equatorial Diameter (EqDiameter), Perimeter Contour, Mean Chord, Length, Width, Min Feret, Max Feret 90, Circularity, Elongation, Shape Factor, Convexity, Roughness, and 3D descriptors of Volume Equatorial Sphere (VolumeEqSphere), Volume Equatorial Cylinder (VolumeEqCylinder). Morphology parameters are created by mathematical operations. They simplified the image data for the subsequent operations. The exact formulas are presented in detail (**S1 Table).** All parameters were calculated as standard functions. The 3D descriptors are specific for the software used. VolumeEqSphere and VolumeEqCylinder are given by Eqs 1 and 2 respectively.


$$VolumeEqSphere=\frac{\pi *{Eqdiameter}^{3}}{6}$$ Eq 1

$$VolumeEqCylinder=\frac{\left(\pi *d^{2})*(l-d\right)}{4}+\frac{\pi *d^{3}}{6}$$ Eq 2

$$Roughness=\frac{Convex\;Hull\;perimeter}{Perimeter}$$ Eq 3

2D descriptor corresponded with surface texture was calculated by Eq 3.

The used colour descriptors included Mean Intensity, Intensity Variation, Mean Red, Mean Green, Mean Blue, Hue Typical, Hue Variation, Mean Saturation, Mean Brightness, Bright Variation, Mean Density, and Density Variation. The colour features are arithmetic mean values on RGB. Hue is converted from RGB to the HSV colour-space. Descriptors were calculated as standard features, for more details see the S1 **(S2 Table)**. And finally, the used extended depth of focus (EDF) descriptors included EDF surface, EDF Roughness, and EDF-Z distance. The automated and semiautomated microscope systems provide EDF image generation function and corresponding 3D reconstruction function by EDF technology. In our study, NIS-Elements EDF reconstruction system (Laboratory Imaging, CZE) was used. The EDF descriptors were simplified according to equations presented in detail. The EDF parameters corresponding to surface texture are EDF Surface and EDF Roughness. They were calculated by Eqs 4 and 5, respectively. EDF-Z distance corresponds to height of the reconstructed object (Eq 6).


$$EdfSurface=\frac{a*b}{2}+\frac{c*d}{2}$$ Eq 4

$$EdfRoughness=\frac{EdfSurface}{Area}$$ Eq 5

$$Mean\;EdfZ=\frac{1}{n}\sum_{n}{EdfZ}_{i}$$ Eq 6

1000 pollen grains were selected in each sample to be classified, and the above mentioned descriptors were measured for them. Following the semi-automated thresholding, pollen grains of dominant taxa in honey samples were classified.

The data were processed statistically using the 2014.5.03 XLSTAT software (Addinsoft, USA). The normality test confirmed the normal distribution of the data. The ANOVA Tukey HSD test was used to compare the pollen characteristics. The multidimensional analysis was provided according to paradigm for object recognition where factor analysis and discrimination analysis are used for object recognition [30].

Factor analysis was used for multi-dimensional description. Factor analysis uses the correlation structure of the measured variables to create “new” variables (factors). The aim is to use the “strong” correlation of the measured variables to reduce the dimensionality of the data while maintaining as much information as possible as well as high level interpretability of the newly created factors. The basis of this method are eigenvalues and eigenvectors of the correlation matrix of the measured variables. The eigenvalues determine the amount of information retained from all the original variables in a given factor, and the corresponding eigenvectors indicate the transformation relationship of the original data to factors. In addition, the product of the square root of the eigenvalue by the corresponding eigenvector gives the correlation of the factors with the original variables. Thus, this procedure produces the same number of factors as the original variables, where each factor is a linear combination of all measured variables. The reduction of the dimension then consists of ignoring the factors with a sufficiently small eigenvalue (usually omitting the factors with an eigenvalue less than 1). Another requirement for a new factor representation is to preserve all factors containing the maximum square of the correlation with some of the original variables.

Discriminant analysis was used to compare the microscopic methods used except for SEM. The aim of the discriminant analysis is to assign the obtained observations based on their measured characteristics to predetermined groups. The idea is to find a data representation that minimizes variability within groups and maximizes variability between groups and then use it for assignment. To perform the discriminant analysis, the decomposition of the covariance matrix into a part corresponding to the intragroup variability and a part corresponding to the intergroup variability is used. Eigenvalues and eigenvectors are again used for the calculation, but this time from the matrix product of the inversion of the intragroup covariance matrix and the intergroup covariance matrix. Again, new variables are created as a combination of all the original variables. The meaning of the eigenvectors is the same as in the factor analysis, but the eigenvalues here express the “discriminatory power” of the resulting variable. The assignment itself then takes place according to the distance from the centres of the predefined groups. The measure of the success of discrimination is then the confusion matrix, where the correct groups are in the rows, the groups assigned using the analysis are in the columns, and inside there is the frequency of each category.

## Results and discussion

### One-dimensional description

Identification of pollen grains by image analysis can be performed using various descriptors. Basic shape characteristics are commonly used [4,6], nevertheless, other characteristics resulting from the textural properties of pollen grains can be utilized as well [19]. For applications other than mellisopalynology, the benefits of EDF for improving discrimination of the objects studied were also confirmed [31]. Another parameter obtainable from the image data is the colour characteristic [32]. A total of 30 descriptors were measured, of which 13 were morphological descriptors, 2 were volume descriptors, 12 were colour descriptors including inner surface texture information, and 3 were EDF descriptors. Shape characteristics are used to describe the geometric parameters of an object and are determined by a simplified mathematical model. For pollen grain discrimination, they represent an important parameter, both, for human evaluation [33] as well as for automatic systems [5,19].

Basic morphological descriptors are often used for pollen identification. Pollen grains are 3D structures which are described by conventional microscopic methods as a plane structure.

The plane description factors are presented in Tables 2 and 5. The important parameter is length which was defined as mean length of the major axis [34], similar to polar axis [35]. The differences in length for evaluated pollen taxa is described in Table 2. The BF shows differences between 4 taxa (p<0.05). The DF shows differences between 5 taxa and Ph shows differences between 5 taxa (p<0.05). The reason is the halo effect around a pollen grain, which enables better automatic detection of object edges. The benefits of the DF technique for pollen identification was also confirmed by several authors [23,36]. Similar number of separated groups was in Ph where object plasticity improved edge detection. The microscopic methods have not been compared to each other in one dimensional description, because different individual pollen grains are analysed. The comparison of our results with two common public atlases (Table 2) showed also differences between atlases and microscopic methods. Statistical analysis was not provided because only mean and SD value are available, and besides, different number of pollen grains was used for these descriptions according to the author’s methodology.

**Table 2:** Comparison of the length of different pollen taxa between microscopy methods and pollen atlases.

	Length (μm)			Pollen atlases	
	BF	DF	Ph	El-Labban, 2020 [37]	Von der Ohe, 2007 [38]
*Asteraceae*	38.14 ±5.28^a^	41.75 ±0.23^e^	62.12 ±5.63^e^	-	32.7±8.1
*Pyrus/prunus*	33.34 ±6.6^a^	35.49 ±6.36^d^	35.37 ±6.2^d^	40.39±6.14	37.41±7.43
*Robinia*	26.67 ±3.67^b^	26.3 ±2.27^c^	26.66 ±2.98^c^	32.5±2.5	30.5±3.2
*Tilia*	26.5 ±2.68^b^	27.71 ±3.84^c^	26.98 ±2.72^c^	32.5±2.5	34.65±1.25
*Brassica*	25.36 ±2.61^b^	25.1 ±2.65^b^	25.12 ±2.29^b^	27.5±2.5	27±2.7
*Salix*	21.58 ±2.98^d^	21.54 ±2.37^a^	20.69 ±2.36^a^	17.5±2.5	18.28±1.95
*Phacelia*	23.59 ±3.98^c^	24.6 ±5^b^	24.69 ±4.6^b^	22.5±2.5	-

Different letter in the column (BF, DF, Ph) indicates significant differences between taxa (p<0.05).

**Fig 2:**
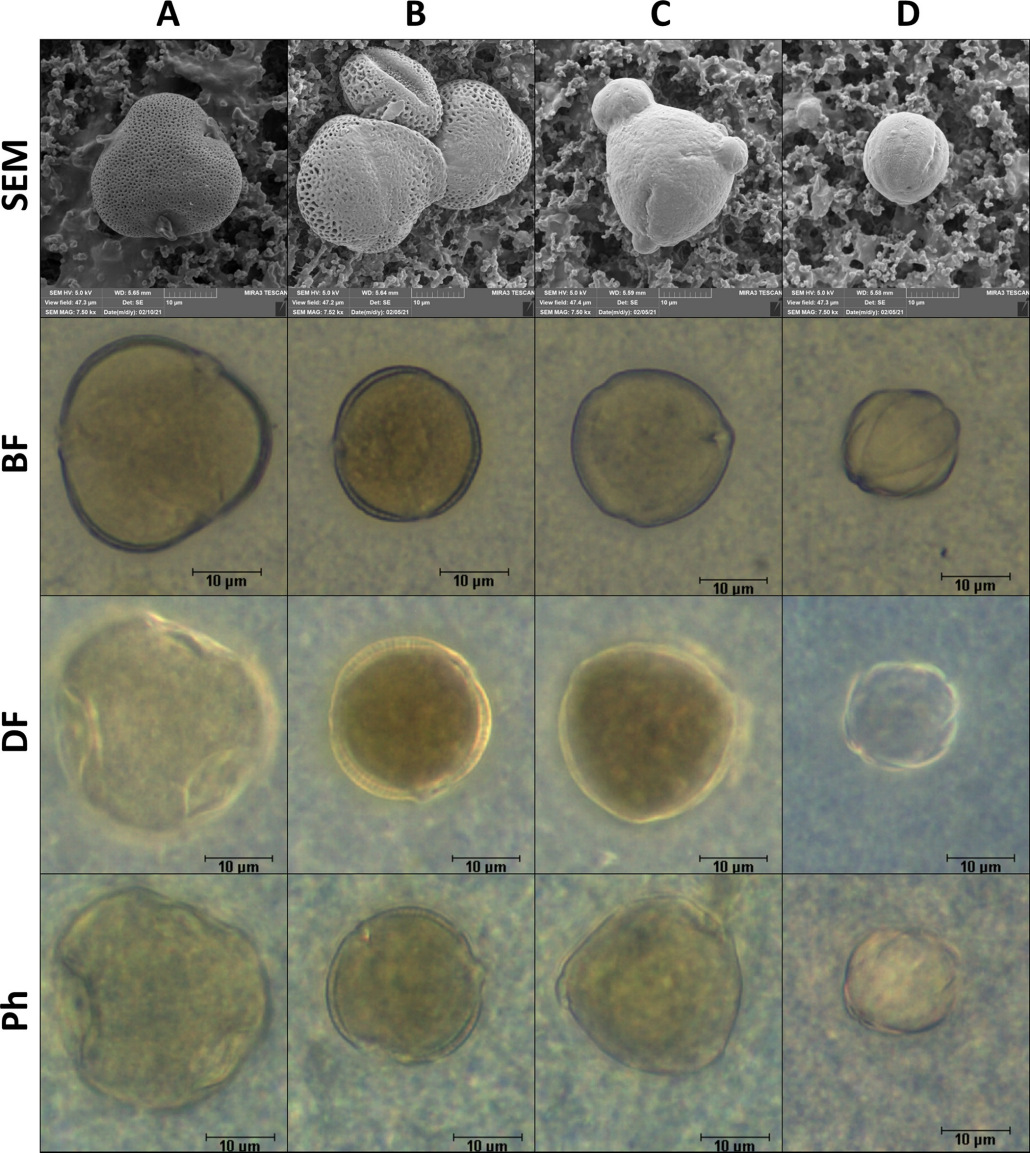
Taxa comparison using microscopic methods, A-*Tilia*; B-*Brassica*; C-*Robinia*; D-*Phacelia*.

Both private databases of pollen grains and publicly available pollen atlases describing characteristic shape properties accompanied by photographs of pollen [1,39] serve as a support for manual evaluation most commonly. So far, private collections [19,32,40], which are then used to discriminate pollen taxa, are used for image analysis. In comparison with other authors, for Ph, the *Brassica* pollen length was 24.48 μm, *Prunus* pollen length was 36.94 μm, *Asteraceae* represents a wide group the pollens sized within the range of 33.16–195.08 [41]. For BF, the size of *Brassica* was measured at 21–30 μm, for *Salix* 16–27 μm, for *Asteraceae* (single species *Helianthus*) 27–31 μm [19], for the *Prunus* 55.15 μm [5]. On the other hand, size and morphological similarities were likely related to the confusion in taxa classification for DF [24]. Size similarity has also been confirmed in our study (Table 2, Fig 2). The morphological characteristics of pollen can differ between botanical species (Fig 2) as well as within botanical species. Morphological differences within a species have been described by several authors and include various reasons for this variability [42]. The length is the common descriptor used for pollen classification based on image processing and manual classification. The length of the pollen can be affected by sample preparation which causes differences between values published [37,38], but also between the results of this study. Another factor that affects pollen grain size is moisture level of the cytoplasm [43]. For the size changing, and important factor is also the type of the apertures which also provide routes for transfer of water and other substances [35]. The aperture can also be used for precise identification of the pollen taxa [44]. This feature was not evaluated in this study, primarily due to the need of a specific algorithm for identification. But apertures have impact on pixel colour, and in our study they are included in colour descriptors. Pollen grain variability ranges considerably and a clear classification according to pollen grain size is not possible in botanical taxa of similar size. Differences in the length measurement of the pollen grain (Table 2). In addition to a similar length of pollen grain in *Tilia* and *Robinia*, both of these botanical taxa are morphologically similar (Fig 2). Therefore, we also assume their similar variability in the spatial arrangement. Likewise, the similarity can be explained for *Brassica* and *Tilia*, where some projections have the same length in planar view. The quantitative technique of filtered grains was used in our study [1], with regard to light scattering, information on the real colour of pollen grains is preserved and the halo effect around pollen grain remains (Fig 2A–2D, DF).

As documented in Fig 2, the image information about the pollen grains on the filters differs from conventional microscopic techniques. The structural as well as colour characteristics of the pollen grains stay, but a lower contrast between the pollen grain and the background is achieved primarily in DF compared to techniques using pollen grain centrifugation. In addition to easier quantification, the speed of this analysis is also an advantage of this filtration technique. However, with regard to the need to make the filter transparent using immersion oil or solacryl, it is necessary to take the morphological changes caused by dehydration, both during drying before mounting and the effect of the organic solvent in the mounting medium. into account. With regard to the stabilizing structures of the pollen wall, these changes are not significant for all taxa.

Other morphological descriptors, some of which have lower impact on the pollen size variability, can be easily calculated also by the image processing. Using the simple geometrical measurement, the shape and ornamentation of pollen can be analysed [32]. The length descriptor as an important parameter was discussed separately (Table 2). The other descriptors used in our study include Area, EqDiameter, Perimeter Contour, Mean Chord, Width, Min Feret, Max Feret 90, Circularity, Elongation, Shape Factor, Convexity, Roughness.

Our results show that circularity, elongation, shape factor, and convexity are not proper for pollen discrimination (Table 3). They divided the pollen taxa into three different groups for Ph, DF, and BF, except for Circularity and Elongation where 2 separate groups were formed for BF, and Elongation with 2 separate groups for DF and Shape Factor with 4 separate groups (Table 3). However, the shape descriptors are not reflected properly in the planar view, namely with regard to the Z-view, which can eliminate hight variability for some botanical taxa [19].

**Table 3:** Shape descriptors of pollen grains.

	Circularity			Elongation			Shape Factor			Convexity		
	BF	DF	Ph	BF	DF	Ph	BF	DF	Ph	BF	DF	Ph
*Asteraceae*	0.85 ±0.02^b^	0.93 ±0.01^b^	0.56 ±0.05^a^	1.12 ±0.05^b^	1.1 ±0.01^a^	1.09 ±0.01^a^	0.93 ±0.01^ab^	0.94 ±0.01^c^	0.91 ±0.01^b^	0.96 ±0^c^	0.97 ±0^b^	0.94 ±0.01^a^
*Pyrus/Prunus*	0.85 ±0.09^b^	0.84 ±0.09^a^	0.87 ±0.08^b^	1.22 ±0.17^b^	1.24 ±0.14^a^	1.26 ±0.16^b^	0.92 ±0.06^b^	0.87 ±0.08^b^	0.89 ±0.07^ab^	0.97 ±0.02^bc^	0.96 ±0.03^a^	0.97 ±0.03^b^
*Tilia*	0.92 ±0.08^a^	0.97 ±0.02^c^	0.97 ±0.02^c^	1.18 ±0.26^b^	1.12 ±0.12^a^	1.1 ±0.06^a^	0.96 ±0.05^a^	0.98 ±0.02^d^	0.98 ±0.01^c^	0.98 ±0.01^a^	0.99 ±0^c^	0.99 ±0^d^
*Robinia*	0.91 ±0.07^a^	0.97 ±0.02^c^	0.95 ±0.04^c^	1.11 ±0.08^b^	1.14 ±0.14^a^	1.11 ±0.04^a^	0.96 ±0.03^a^	0.98 ±0.02^d^	0.97 ±0.01^c^	0.98 ±0.01^ab^	0.99 ±0^c^	0.98 ±0.01^c^
*Brassica*	0.93 ±0.05^a^	0.97 ±0.03^c^	0.93 ±0.06^c^	1.17 ±0.13^b^	1.16 ±0.13^a^	1.15 ±0.12^ab^	0.97 ±0.02^a^	0.98 ±0.02^d^	0.97 ±0.02^c^	0.98 ±0.01^a^	0.99 ±0^c^	0.98 ±0.01^c^
*Salix*	0.91 ±0.05^a^	0.95 ±0.04^c^	0.95 ±0.04^c^	1.17 ±0.13^b^	1.2 ±0.14^a^	1.22 ±0.14^ab^	0.96 ±0.02^a^	0.96 ±0.03^cd^	0.96 ±0.02^c^	0.98 ±0.01^ab^	0.99 ±0.01^c^	0.99 ±0.01^cd^
*Phacelia*	0.81 ±0.07^b^	0.82 ±0.06^a^	0.85 ±0.07^b^	1.82 ±0.38^a^	2 ±0.3^b^	1.81 ±0.36^c^	0.85 ±0.07^c^	0.83 ±0.06^a^	0.86 ±0.07^a^	0.98 ±0.01^ab^	0.99 ±0.01^c^	0.99 ±0.01^d^

Different letter in the column indicates significant differences between taxa (p<0.05).

The shape descriptors of Circularity, Elongation, Shape Factor, and Convexity describe specific characteristics regarding the geometry of a pollen grain. These shape descriptors are invariant with respect to translation, rotation, and scaling. These measurements are clearly defined, relatively easy to use and there is a fast and easily understood procedure for their computing [45]. Therefore, these parameters specify the discrimination of individual pollen grains and are also used in descriptive palynological studies. Common pollen shapes are circular, elliptic, lobate, triangular, and polygonal [46], which are described in our study by the parameter of Circularity. The Elongation is more representative for common description of the pollen terminology know as P/E (polar axis/equatorial axis). P/E is widely used system for a shape classes definition. For the spheroidal pollen in range of 0.88–1.14, for suboblate 0.75–0.88 and for subprolate 1.14–1.33 [35]. The Shape Factor described object roughness. It is based on morphometric measurement, this characteristic represented pollen ornamentation. In the morphometric principle it is able to detect spine only in distances according to the resolution of the microsystems used. Other morphological descriptors show higher ability to divide taxa into separate groups (Table 4). These descriptors are size dependent and they provided basic information of the pollen shape [32].

**Table 4:** Size depended morphological descriptors.

	Area (μm^2^)			EqDiameter (μm)			Perimeter Contour (μm)			Mean Chord (μm)		
	BF	DF	Ph	BF	DF	Ph	BF	DF	Ph	BF	DF	Ph
*Asteraceae*	1010.36 ±298.59^a^	1175.94 ±29.51^f^	1144.48 ±16.93^e^	35.37 ±5.95^a^	38.69 ±0.49^f^	38.17 ±0.28^e^	122.37 ±21.5^a^	127.67 ±1.7^f^	163.77 ±9.37^f^	25.56 ±4.15^a^	29.31 ±0.44^f^	22.33 ±0.87^d^
*Pyrus/Prunus*	680.57 ±218.79^b^	743.27 ±213.18^e^	745.81 ±214.2^d^	29.07 ±4.61^b^	30.46 ±4.34^e^	30.51 ±4.33^d^	100.42 ±17.78^b^	105.98 ±17.85^e^	104.68 ±17.26^e^	21.05 ±3.39^b^	21.84 ±3.03^e^	22.23 ±3.08^d^
*Robinia*	490.11 ±124.4^c^	478.6 ±69.08^c^	500.62 ±109.91^c^	24.75 ±3.36^c^	24.63 ±1.72^c^	25.11 ±2.66^c^	81.84 ±11.02^c^	78.9 ±5.59^c^	80.56 ±8.74^d^	18.69 ±2.81^c^	19.08 ±1.35^d^	19.45 ±2.03^c^
*Tilia*	478.12 ±88.57^cd^	532.52 ±141.79^d^	507.08 ±109.22^c^	24.57 ±2.24^cd^	25.82 ±3.37^d^	25.27 ±2.61^c^	81.97 ±8.37^c^	82.82 ±10.85^d^	83.14 ±8.52^d^	18.38 ±1.81^cd^	20 ±2.64^c^	19.39 ±2.18^c^
*Brassica*	424.08 ±61.33^d^	421.49 ±66.71^b^	421.92 ±52.91^b^	23.18 ±1.67^d^	23.1 ±1.8^b^	23.13 ±1.45^b^	76.44 ±6.15^d^	74.27 ±6.09^b^	76.34 ±5.68^c^	17.52 ±1.29^d^	17.84 ±1.37^b^	17.49 ±1.19^b^
*Salix*	304.8 ±85.71^e^	299.44 ±61.7^a^	272.05 ±59.54^a^	19.53 ±2.57^e^	19.42 ±2.05^a^	18.5 ±2.05^a^	64.94 ±9.16^e^	63.03 ±6.77^a^	60.25 ±7.07^a^	14.63 ±1.92^e^	14.84 ±1.62^a^	14.13 ±1.53^a^
*Phacelia*	287.8 ±73.49^e^	285.17 ±84.78^a^	287.77 ±77.94^a^	18.97 ±2.54^e^	18.83 ±2.9^a^	18.96 ±2.6^a^	67.19 ±9.75^e^	65.82 ±10.06^a^	65.07 ±9.37^b^	13.36 ±1.8^f^	13.39 ±2.19^a^	13.73 ±1.94^a^

Different letter in the column indicates significant differences between taxa (p<0.05).

For BF and Ph, pollen was classified into five groups for Area, EqDiameter, and Perimeter Contour. In contrast, for DF all taxa were classified into 6 different groups (p <0.05). The Mean Chord shows the variable differences of taxa groups in microscopic methods comparison. DF divided taxa to 6 groups, BF to 5 groups, and Ph to 4 groups. The Equatorial Diameter is a parameter used also for pollen characterisation by human evaluation. In comparison, EqDiamether is slightly different because it is calculated as a diameter of a circle with the same area as the measured object (S1 Table), not as a line, lying in the equatorial plane [35].

The results are in conformity with other authors who confirmed DF as a suitable method for pollen discrimination [23,47]. The chord length describes object shape which is represented by a set of n-vertex polygons [48]. In our study, it is characterised as a mean value measured at 0, 45, 90 and 135 degrees directions (**S1 Table**). The chord length is highly relevant to the properties related to heterogeneous materials [49,50], in conformity with these, our results confirmed that it is also usable for pollen classification in BF and DF.

The complementary morphological descriptors were able to classify the pollen taxa into separate groups. Six different groups were formed by Width, Min Feret, and Max Feret 90 descriptors for BF and DF (p <0.05). Ph reached a lower number of separate groups for Width and Min Feret, nevertheless, MaxFeret90 separated six different groups similarly to BF and DF (Table 5). Our results confirmed that these descriptors can be used for classification and they show slight differences between individual microscopic methods. Feret diameter is also confirmed in literature as a relevant descriptor for pollen classification by DF [51] and by BF [5]. P/E ratio is commonly used for pollen description, where P stands for polar diameter and E stands for equatorial diameter. In our morphometric characterization, it is represented by Elongation (Max Feret 90/Min Feret) but spatial orientation of the microspore is not reflected. The Width is a complementary descriptor to the Length. It is appropriated for elongated pollen taxa and for dried pollen. The useful length descriptors for particle size classification are also Min Feret and Max Feret 90 and they can be also use for description of object elongation [52]. The Max Feret 90 is a complementary parameter with equatorial diameter used for pollen characterisation in pollen terminology [35].

**Table 5:** Complementary morphological descriptors.

	Width (μm)			Min Feret (μm)			Max Feret 90 (μm)		
	BF	DF	Ph	BF	DF	Ph	BF	DF	Ph
*Asteraceae*	25.79 ±5.07^f^	28.17 ±0.77^f^	18.55 ±1.4^c^	34.49 ±6.12^f^	37.74 ±0.28^f^	41.16 ±0.39^d^	35.96 ±6.73^f^	39.99 ±0.68^f^	37.96 ±0.73^f^
*Pyrus/Prunus*	20.06 ±3.27^e^	20.68 ±3.19^e^	20.8 ±3.14^d^	27.49 ±4.34^e^	29.33 ±4.77^e^	35.95 ±6.11^d^	28.26 ±4.54^e^	30.21 ±4.95^e^	28.82 ±4.65^e^
*Robinia*	18.13 ±2.93^d^	18.15 ±1.49^d^	18.58 ±2.03^c^	23.5 ±3.9^d^	23.56 ±1.8^d^	26.66 ±2.98^c^	23.98 ±3.97^d^	24.21 ±1.93^d^	24.24 ±2.59^d^
*Tilia*	17.94 ±1.95^d^	18.94 ±2.56^d^	18.61 ±2.04^c^	23.78 ±2.29^d^	24.43 ±3.39^d^	26.98 ±2.72^c^	24.65 ±2.45^d^	25.15 ±3.52^d^	24.44 ±2.63^d^
*Brassica*	16.69 ±1.34^c^	16.74 ±1.45^c^	16.78 ±1.25^b^	21.88 ±1.71^c^	21.77 ±1.83^c^	25.16 ±2.28^b^	22.28 ±1.77^c^	22.3 ±1.91^c^	21.98 ±1.58^c^
*Salix*	13.94 ±2.17^b^	13.8 ±1.83^b^	13.04 ±1.78^a^	18.46 ±2.72^b^	18.13 ±2.31^b^	20.71 ±2.37^a^	18.89 ±2.88^b^	18.67 ±2.46^b^	17.14 ±2.28^b^
*Phacelia*	12.15 ±2.34^a^	11.52 ±2.37^a^	11.57 ±1.88^a^	14.62 ±2.34^a^	13.67 ±2.55^a^	25.91 ±4.54^b^^c^	14.8 ±2.37^a^	13.86 ±2.52^a^	14.5 ±2.26^a^

Different letter in the column indicates significant differences between taxa (p<0.05).

The 3D descriptor was able to divide the pollen taxa to separate groups depending on the microscopic technique used (Table 6). For DF, the taxa were divided into 6 groups, for BF and Ph, they were classified into 5 significant different groups (p <0.05). 3D discrimination for pollen taxa was confirmed for BF [40,53,54] where various characteristic were used. Our results confirmed that 3D reconstruction can be used for discrimination of pollen taxa also for DF and Ph, where differences between taxa were found (p <0.05).

**Table 6:** Volume descriptors.

	VolumeEqSphere (μm^3^)			VolumeEqCylinder (μm^3^)		
	BF	DF	Ph	BF	DF	Ph
*Asteraceae*	25008.7 ±9682.52^a^	30342.1 ±1139.19^f^	29128.17 ±647.67^e^	16682.26 ±6571.83^a^	20169.13 ±877.25^f^	14960.25 ±708.37^e^
*Pyrus/Prunus*	13860.17 ±6818.67^b^	15704.87 ±6842.05^e^	15786.02 ±6905.51^d^	9157.65 ±4684^b^	10446.01 ±4781.89^e^	10424.96 ±4673.98^d^
*Robinia*	8363.28 ±3106.08^c^	7936.04 ±1786.02^c^	8573.61 ±2932.24^c^	5686.08 ±2149.53^c^	5293.68 ±1203.06^c^	5732.5 ±1954.6^c^
*Tilia*	7963.97 ±2257.87^c^^d^	9483.78 ±3879.13^d^	8733.98 ±2949.11^c^	5292.34 ±1516.85^c^^d^	6312.9 ±2563.51^d^	5832.49 ±1975.59^c^
*Brassica*	6620.79 ±1449.94^d^	6569.32 ±1620.67^b^	6557.9 ±1233.99^b^	4375.56 ±943.83^d^	4346.71 ±1052.6^b^	4353.41 ±819.23^b^
*Salix*	4114.5 ±1909.42^e^	3960.46 ±1199.53^a^	3436.1 ±1114.77^a^	2707.37 ±1270.08^e^	2601.91 ±815.6^a^	2253.13 ±755.84^a^
*Phacelia*	3764.48 ±1382.61^e^	3743.42 ±1633.23^a^	3772.12 ±1547.15^a^	2642.2 ±1212.37^e^	2447.81 ±1178.64^a^	2363.79 ±996.4^a^

Different letter in the column indicates significant differences between taxa (p<0.05).

As already mentioned before, pollen grains are 3D structures. Various methods for 3D acquisition and visualization of pollen was used [40,53,54]. 3D descriptors in our study are calculated from several focal planes. The descriptor used was VolumeEqSphere, and VolumeEqCylinder and they represented a simplified 3D model of irregular pollen shape (Table 6). This model is used for size characterization of a particle in 3D reconstructed objects [55].

Important factor for pollen determination by image processing is the surface of the pollen grain [6,21] formed by the pollen wall. In our study, this descriptor is based on colour and shape characteristics. A comparison of the differences within the observed taxa is summarised in Table 7. EDF Surface divided pollen into the highest number of groups, BF, DF, and Ph show 5, 6, and 3 separate groups (p <0.05), respectively. Roughness assigned the taxa into 4, 3, 2 separate groups for Ph, DF, and BF, (p <0.05), respectively. And finally, EDF Roughness separated the taxa into 1, 3, and 3 (p <0.05) groups for BF, DF and Ph, respectively. Our results show low variability between taxa for this descriptor. BF, DF, and Ph separate groups within taxa reached 3, 2, 2 respectively (Table 7).

**Table 7:** Roughness and EDF descriptors.

		Roughness			EDF Surface			EDF Roughness			Edf-Z (μm)		
Exine	Taxa	BF	DF	Ph	BF	DF	Ph	BF	DF	Ph	BF	DF	Ph
Echinate	*Asteraceae*	0,95 ±0,01^a^	0,99 ±0^b^	0,78 ±0,03^a^	1443,05 ±613,87^e^	2655,49 ±666,26^f^	2715,11 ±345,82^b^^c^	1,38 ±0,32^a^	2,26 ±0,55^c^	2,37 ±0,29^a^	5,27 ±2,78^a^	9,81 ±2,61^a^^b^	20,08 ±2,34^b^
Striate	*Pyrus/Prunus*	0,97 ±0,03^a^^b^	0,98 ±0,02^a^	0,99 ±0,02^c^	963,98 ±319,21^d^	1157,8 ±426,55^e^	2117,37 ±2461,86^a^^b^	1,42 ±0,15^a^	1,55 ±0,3^b^	3,32 ±4,67^a^^b^	11,07 ±2,53^c^	11,68 ±3,77^a^^b^	10,27 ±4,17^a^
Psilate	*Robinia*	0,98 ±0,03^b^	1 ±0^b^	1 ±0^d^	746,91 ±203,83^c^	730,03 ±134,77^c^	4357,29 ±3303,96^c^	1,52 ±0,16^a^	1,52 ±0,18^a^^b^	8,59 ±6,22^c^	10,46 ±2,45^b^^c^	9,01 ±2,4^a^	15,17 ±5,81^a^^b^
Reticulate	*Tilia*	0,97 ±0,02^a^^b^	1 ±0^b^	0,99 ±0,02^c^^d^	667,22 ±153,71^b^^c^	1044,69 ±419,97^d^	851,52 ±265,39^a^	1,39 ±0,13^a^	1,92 ±0,38^c^	1,67 ±0,31^a^	6,75 ±2,3^a^	13,22 ±3,26^b^	10,63 ±2,06^a^
Reticulate	*Brassica*	0,98 ±0,02^b^	0,99 ±0,01^b^	0,98 ±0,03^b^	593,86 ±114,86^b^	646,2 ±165^b^	992,46 ±1437,4^a^	1,4 ±0,15^a^	1,53 ±0,29^b^	2,36 ±3,45^a^	10,78 ±3,02^b^^c^	12,42 ±4,09^a^^b^	12,12 ±6,1^a^
Reticulate	*Salix*	0,97 ±0,02^b^	0,99 ±0,01^b^	0,99 ±0,01^c^^d^	428,89 ±130,13^a^	440,55 ±128,02^a^	997,49 ±1402,89^a^	1,41 ±0,16^a^	1,47 ±0,29^a^^b^	3,89 ±5,47^a^^b^	10,18 ±2,81^b^	11,76 ±4,64^a^^b^	13 ±5,37^a^
Micro- reticulate	*Phacelia*	0,97 ±0,02^b^	1 ±0^b^	0,99 ±0^d^	426,88 ±122,97^a^	415,03 ±160,3^a^	2104,09 ±2741,91^a^^b^	1,48 ±0,22^a^	1,43 ±0,22^a^	7,32 ±8,19^b^^c^	10,36 ±2,14^b^^c^	9,73 ±2,74^a^^b^	13,26 ±3,78^a^

Different letter in the column indicates significant differences between taxa (p<0.05).

Colour characteristic represented textural information calculated as the spatial organisation of RGB levels of an image (Mean Brightnes, Intensity Variation, and Bright Variation). The shape characteristics represented textural information on Roughness, EDF Surface and EDF Roughness of pollen grains which correspond to the exine layer. In particular, the outer exine layer–sexine–creates various arrangements that form the typical pollen surface (columellae, tectum, supratectal elements) [56]. In 2D, the exine sculpture is most characterized by the morphometric parameter of Roughness and in 3D by EDF Surface and EDF Roughness.

**Fig 3:**
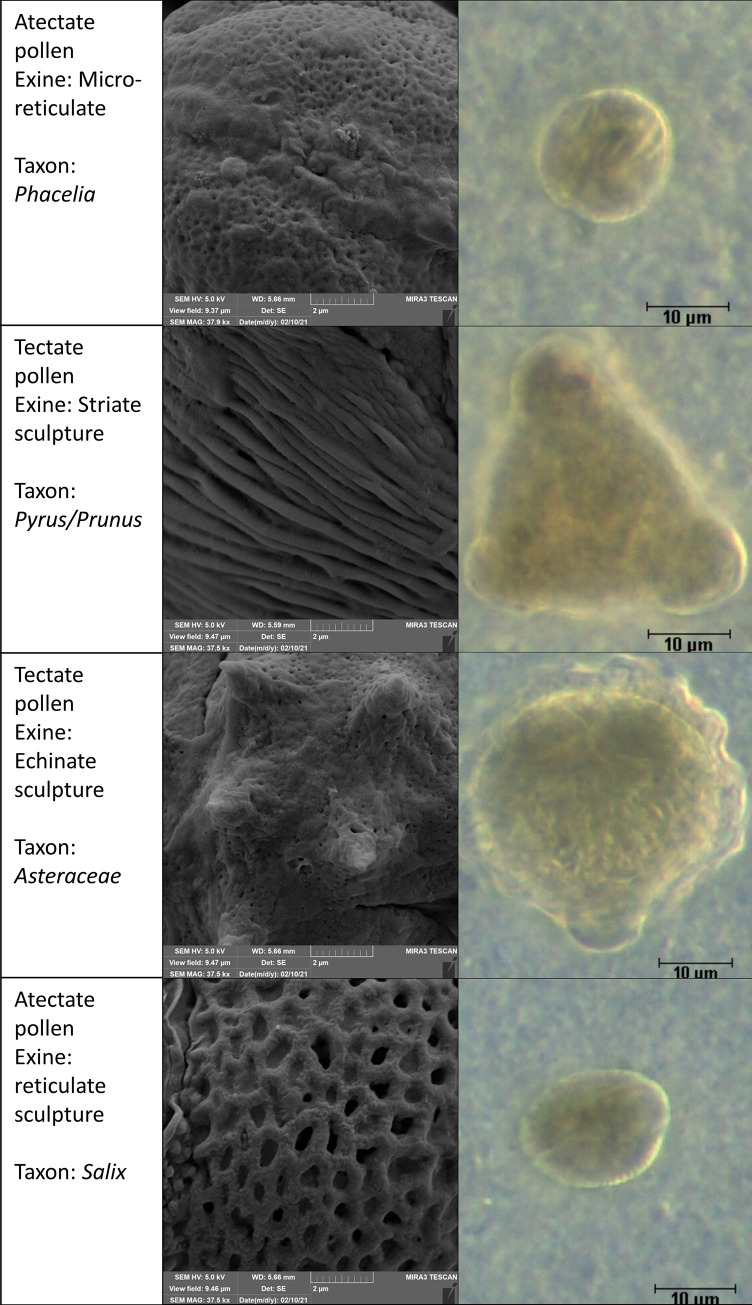
Typical exine sculptures of the botanical taxa studied, left–SEM, right–DF.

Exact correspondence with the exine sculpture, however, was not demonstrated for either parameter. This is because the accurate exine visualization can only be achieved at higher resolutions, typically using SEM microscopic techniques [57]. In light microscopy, the differences cannot be detected with high accuracy. Sometimes, there is even a different classification of exine sculpture depending on the microscopic technique used. Typical exine sculptures are shown in Fig 3. The suitability of surface texture for pollen identification was also confirmed [6]. EDF derivate descriptor without texture correspondence is EDF-Z, these represented mean distance in reconstructed Z-view. This distance is complementary to the length of the pollen grain but in the direction towards the optical view.

The results of pixel brightness, hue, and pixel distribution are summarised in Table 8. The Mean Brightness, Intensity Variation, and Bright Variation divided the taxa into 4, 3, and 5 groups (p <0.05) for BF, DF, and Ph, respectively. Hue Typical represented the colour of the pollen and it divided the taxa into 4, 5, 4 separate groups (p <0.05) for BF, DF, and Ph, respectively. Surface texture of a pollen grain affects the distribution of pixel brightness values of the observed object. This distribution can be characterized by various parameters (Mean Value, Coefficient of Variation, Askewness and Kurtosis). The Mean Value and Coefficient of Variation were used in our study. The coefficient of Variation including Intensity and Bright Variation. These descriptors represented characteristic elements on pollen surface [58]. Notably, Lacuna, aperture, annulus, arcus, atrium, sculpture elements, tectum, and columella are used for precise identification of pollen taxa by human evaluators. In our study, variation value characterises all surface elements based on differences between pixel intensity and brightness.

**Table 8:** Texture related colour descriptors of pollen grains.

	Mean Brightness			Intensity Variation			Bright Variation			Hue Typical		
	BF	DF	Ph	BF	DF	Ph	BF	DF	Ph	BF	DF	Ph
*Asteraceae*	45.45 ±2.3^d^	54.23 ±0.39^b^^c^	38.02 ±0.25^b^^c^^d^	12.31 ±0.25^d^	23.8 ±0.58^c^	10.2 ±0.34^b^^c^^d^	4.83 ±0.1^d^	9.33 ±0.23^c^	4 ±0.14^b^^c^^d^	32.05 ±0.17^a^	47.04 ±1.28^b^^c^	33.74 ±0.16^a^
*Pyrus/Prunus*	42.68 ±5.65^c^^d^	52.48 ±4.74^b^^c^	43.37 ±4.12^c^^d^	10.41 ±2.11^d^	16.6 ±3.59^a^^b^	10.75 ±1.43^c^^d^	4.08 ±0.83^d^	6.51 ±1.41^a^^b^	4.22 ±0.56^c^^d^	38.79 ±4.04^c^	52.69 ±15.76^c^	39.52 ±4.44^b^
*Robinia*	35.33 ±1.37^a^	55.11 ±3.43^c^	49.72 ±2.74^e^	8.16 ±1.46^a^^b^	18.57 ±3.37^b^	13.51 ±3.24^e^	3.2 ±0.57^a^^b^	7.28 ±1.32^b^	5.3 ±1.27^e^	43.9 ±3.25^d^	73.54 ±9.89^d^	41.09 ±2.08^b^
*Tilia*	44.13 ±3.09^c^^d^	47.11 ±4.09^a^	41.94 ±2.89^a^^b^	9.9 ±1.47^c^^d^	23.51 ±4.39^c^	8.91 ±1.03^a^^b^	3.88 ±0.57^c^^d^	9.22 ±1.72^c^	3.49 ±0.41^a^^b^	33.4 ±3.28^a^^b^	37.64 ±2.92^a^	46.48 ±2.29^c^
*Brassica*	38.14 ±4.48^a^^b^	50.67 ±3.8^a^^b^	37.15 ±2.09^a^	8.93 ±1.66^b^^c^	17.78 ±4.13^b^	8.63 ±1.32^a^	3.5 ±0.65^b^^c^	6.97 ±1.62^b^	3.38 ±0.52^a^	34.82 ±3.35^a^^b^	43.76 ±6.5^a^^b^	38.22 ±4.82^a^^b^
*Salix*	40.37 ±5.83^b^^c^	53.16 ±4.42^b^^c^	43.18 ±5.3^a^^b^^c^	9.95 ±4.46^d^	17.21 ±4.26^a^^b^	9.51 ±1.84^a^^b^^c^	3.9 ±1.75^d^	6.75 ±1.67^a^^b^	3.73 ±0.72^a^^b^^c^	35.54 ±4.19^b^	47.15 ±8.88^b^^c^	39.98 ±5.94^b^
*Phacelia*	36.31 ±1.35^a^^b^	55.68 ±2.93^c^	50.39 ±2.4^d^	7.36 ±1.47^a^	13.87 ±3.1^a^	11.14 ±1.82^d^	2.89 ±0.57^a^	5.44 ±1.21^a^	4.37 ±0.71^d^	40 ±2.74^c^	95.22 ±20.68^e^	42.69 ±7.83^b^^c^

Different letter in the column indicates significant differences between taxa (p<0.05).

The use of colour characteristics to identify and discriminate microscopic objects has been validated in several studies [32,40,53,59,60]. Although colour characteristics are important for pollen grain discrimination, their transfer to other systems is difficult. The reason is the influence of the light source, microscopic glass, microscope optics, RGB sensor, and the influence of post-processor processing on the colour of the digital image [61]. For proper transferability, the optical system of the microscope as well as camera parameters must allow accurate standardization of the device [62]. In our study, only one optical system and one camera were used, and for each measurement, the microscopic system was set to the reference spectrum using a spectrophotometer as a standard.

In our study, RGB colour descriptors confirm significant differences between taxa (p <0.05) for microscopic methods (Table 9). Mean value of RGB and Mean Density were used as colour descriptors. The highest variability between taxa was confirmed for Ph. Different groups for Mean Red and Mean Green reached 3, for Mean Blue reached 5 (p <0.05). The BF shows 3 different groups for Mean Red and Mean Blue, 4 for Mean Green (p <0.05). The DF shows 3 different groups for Mean Blue and 2 groups for Mean Red and Mean Blue (p <0.05). The derivate function for RGB is Mean Density. Mean Density shows the highest variability for Ph (5 groups) and similar for BF and DF (3 groups). In contrast to [63], where BF was used and only Mean Red was relevant, our results show that Mean Blue and Mean Green colour descriptors can be relevant for pollen classification depending on the microscopic method used. This reflects human perception, where the RGB colour system is used. By red–green–blue combination, every possible colour is defined [59].

**Table 9:** RGB colour descriptor of pollen grains.

	Mean Red			Mean Green			Mean Blue			Mean Density		
	BF	DF	Ph	BF	DF	Ph	BF	DF	Ph	BF	DF	Ph
*Asteraceae*	137.52 ±4.9^d^	147.75 ±0.72^b^	110.71 ±1.09^a^^b^	122.58 ±6.09^d^	148.61 ±0.98^b^	102.45 ±0.79^a^	87.58 ±6.96^c^	118.48 ±1.53^b^	77.73 ±0.29^a^^b^	0.36 ±0.02^a^	0.27 ±0^a^^b^	0.43 ±0^d^^e^
*Pyrus/Prunus*	123.66 ±17.09^b^^c^	141.14 ±7.84^a^^b^	122.65 ±10.86^c^	115.26 ±14.85^c^^d^	142.29 ±11.77^b^	117.74 ±10.3^b^	87.57 ±12.16^c^	118.02 ±18.11^b^	91.39 ±10.9^d^	0.38 ±0.06^a^^b^	0.29 ±0.05^a^^b^	0.37 ±0.04^b^
*Robinia*	99.88 ±3.08^a^	141.26 ±9.15^a^^b^	139.67 ±7.8^d^	95.94 ±3.69^a^	147.49 ±8.94^b^	133.33 ±7.09^c^	74.48 ±4.23^a^	132.81 ±8.71^c^	107.37 ±6.34^e^	0.46 ±0.02^c^	0.26 ±0.03^a^	0.31 ±0.02^a^
*Tilia*	134.81 ±7.16^c^^d^	135.01 ±8.47^a^	116.44 ±7.24^b^^c^	118.4 ±8.58^c^^d^	129.21 ±10.81^a^	117.27 ±8.13^d^	84.42 ±8.52^b^^c^	96.15 ±12.44^a^	87.12 ±7.09^b^^c^	0.37 ±0.03^a^^b^	0.34 ±0.04^c^	0.39 ±0.03^c^^d^
*Brassica*	116.12 ±13.33^b^	141.64 ±6.83^a^^b^	107.31 ±6.26^a^	103.63 ±11.84^a^^b^	140.79 ±9.98^b^	101.93 ±5.7^a^	72.05 ±9.84^a^	105.18 ±13.65^a^^b^	74.95 ±5.24^a^	0.43 ±0.06^c^	0.31 ±0.04^b^^c^	0.44 ±0.03^e^
*Salix*	120.03 ±16.11^b^	146.39 ±9.56^b^	122.54 ±13.71^c^	109.24 ±14.94^b^^c^	147.35 ±11.98^b^	118.36 ±14.91^b^	79.59 ±14.85^a^^b^^c^	112.93 ±15.08^b^	89.45 ±12.69^c^^d^	0.41 ±0.07^b^^c^	0.28 ±0.04^a^^b^	0.37 ±0.06^b^^c^
*Phacelia*	101.42 ±3.12^a^	139.1 ±9.52^a^	139.44 ±8.49^d^	98.23 ±3.66^a^	148.15 ±8.12^b^	135.94 ±5.98^c^	78.12 ±4.14^a^^b^	138.66 ±8.85^c^	110.09 ±6.29^e^	0.45 ±0.02^c^	0.26 ±0.02^a^	0.3 ±0.02^a^

Different letter in the column indicates significant differences within taxa (p <0.05).

Transferred RGB colour space in to the HSB (hue, saturation, brightness) colour space is shown in Table 10. Our results show that Hue Variation had a lower variability between taxa in BF. For DF and Ph, the taxa were separated into 4 different groups (p <0.05) (Table 10). Mean Saturation was more variable between the taxa. For BF, the taxa were separated to 5 different groups, for DF and Ph into 4 different groups (p <0.05). The brightness represented also textural features as described above (Table 8). The mean intensity classified taxa for Ph, into 5 groups, for BF into 4 groups, and for DF into 3 different groups (p<0.05). Mean Intensity was used for pollen classification using autofluorescence image analysis [64]. HSB colour space was successfully used for pollen discrimination in BF [63]. Our results show that other microscopic methods can be used as well (Table 10).

**Table 10:** Intensity and HSB colour descriptors of pollen grains.

	Mean Intensity			Hue Variation			Mean Saturation		
	BF	DF	Ph	BF	DF	Ph	BF	DF	Ph
***Asteraceae***	115.9 ±5.87^d^	138.28 ±0.98^b^^c^	96.96 ±0.64^a^^b^	18.16 ±2.55^a^	15.14 ±1.3^c^	11 ±1.09^a^	62.09 ±5.97^d^^e^	37 ±1.37^b^^c^	50.06 ±1.37^c^^d^
***Pyrus/Prunus***	108.83 ±14.4^c^^d^	133.82 ±12.08^b^^c^	110.59 ±10.52^d^	25.21 ±7.56^a^^b^	14.95 ±8.96^b^^c^	16.16 ±5.32^b^^c^	49.94 ±8.39^c^	32.2 ±16.08^b^	44.43 ±6.39^b^
***Robinia***	90.1 ±3.49^a^	140.52 ±8.74^c^	126.79 ±6.98^e^	30.48 ±7.48^b^	30.58 ±4.17^d^	25.77 ±5.19^d^	44.73 ±5.31^b^	16.51 ±2.24^a^	38.59 ±2.99^a^
***Tilia***	112.54 ±7.88^c^^d^	120.13 ±10.42^a^	106.95 ±7.37^b^^c^	26.04 ±9.76^a^^b^	6.4 ±1.75^a^	14.34 ±2.93^a^^b^^c^	64.62 ±8.02^e^	51.94 ±10.43^d^	47.38 ±4.63^b^^c^
***Brassica***	97.27 ±11.41^a^^b^	129.2 ±9.7^a^^b^	94.73 ±5.33^a^	22.54 ±9.88^a^^b^	7.74 ±3.57^a^	12.35 ±4.04^a^^b^	66.54 ±7.91^e^	48.07 ±13.27^c^^d^	53.23 ±5.42^d^
***Salix***	102.95 ±14.86^b^^c^	135.56 ±11.27^b^^c^	110.12 ±13.51^c^^d^	21.86 ±11.91^a^	10.45 ±6.43^a^^b^	11.96 ±4.37^a^^b^	58.5 ±12.57^d^	43.07 ±14.37^b^^c^^d^	47.69 ±6.83^b^^c^^d^
***Phacelia***	92.59 ±3.45^a^^b^	141.97 ±7.46^c^	128.49 ±6.11^e^	20.81 ±8.03^a^	32.3 ±7.59^d^	18.29 ±6.14^c^	39.53 ±5.17^a^	16.57 ±6.92^a^	36.04 ±7.52^a^

Different letter in the column indicates significant differences between taxa (p <0.05).

The sequence of the measured descriptors for morphological and colour characteristics of pollen has been described above. However, none of the descriptors allows clear identification of the studied taxa in any microscopic methods used. In the case of multiple descriptors, use of discriminant analyses is the most appropriate, which has also been successfully validated for pollen discrimination by a number of authors [4,19,21,24,60].

It can also be assumed that the more taxa studied, the more characteristics will be necessary for the recognition. In our study, honey from the Czech Republic was used, which is typically affected by large-scale farming [28]. In comparison, honey can be collected also from country with fragmented agricultural activity [65], from national parks [66,67] or other region with high floral diversity, where typically one family is represented by multiple species, wild cultivars and endemism. These types of honey are also typical by high content of pollen with similar shape, polar and equatorial length, P/E, and pollen ornamentation. In these types of honey, the precise identification should be provided. In general, identification of the type of surface, lacune, apertures, thickness of apertures, ornamentation is provided often by means of a combination of different microscopic techniques.

### Multi-dimensional description

**Fig 4:**
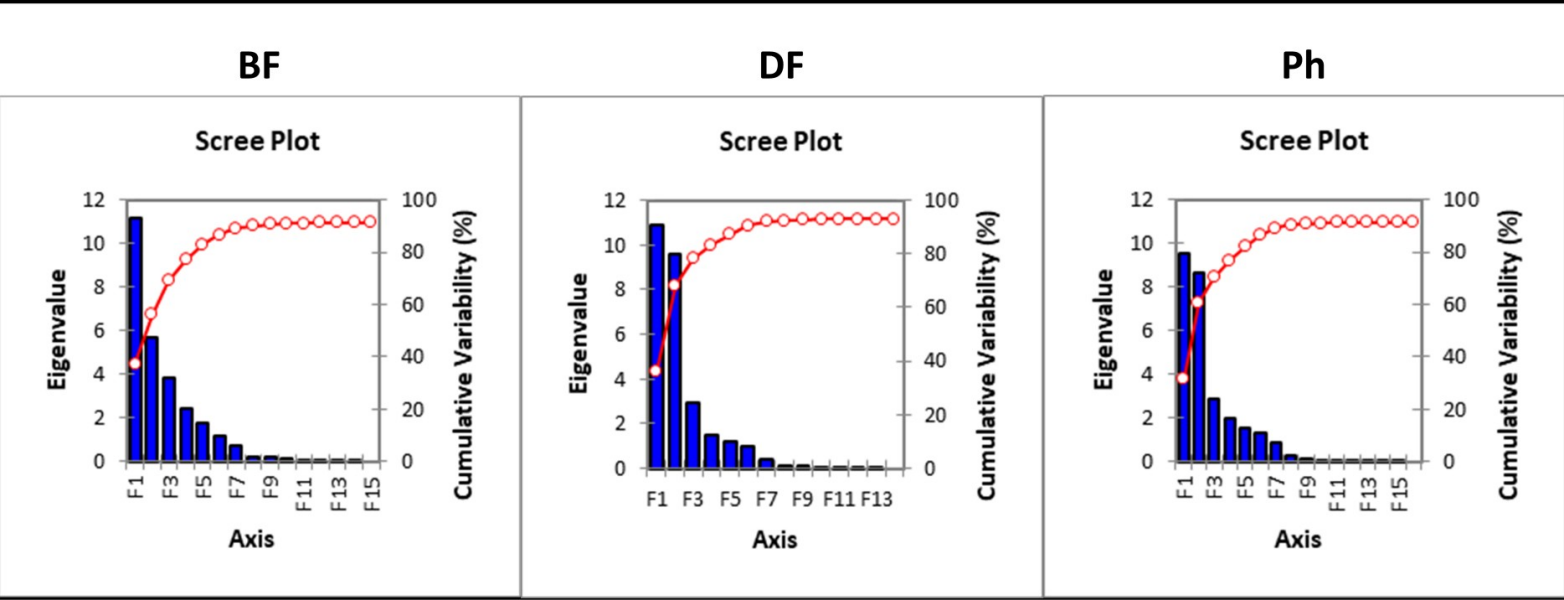
Scree plots of factor analysis for different microscopic techniques (BF-bright field, DF-dark field, Ph-phase contrast).

Due to the number of characteristics measured and the dependence of some of them, it is possible to reduce the size of the input data before. This was done by factor analysis. Already the first two BF factors retain 56.26% of the information contained in the original thirty characteristics (68.35% for DF and 60.67% for Ph). In all cases (BD, DF, and Ph), all factors corresponding to eigenvalues greater than 1 and all factors containing the maximum of the squared correlation with one of the original variables were used for the discriminant analysis (**S3 Table. Factor variable correlation matrix**). Due to the size of the eigenvalues relevant to other factors, the information from them can be declared insignificant and can be considered “as noise”. The first 6 factors retaining 91.1% of the original information were used for discriminant analysis of the data obtained by DF; and 7 factors retaining 89.23% and 89.09% information for DF and BF, respectively. The results of the factor analysis are illustrated by the scree plots in Fig 4.

Factor 1 for BF consists mainly of morphometric parameters (**S3 Table**). The measured morphometric parameters can be divided into 2D characteristics (Area, EqDiameter, Perimeter Contour, Mean Chord, Length, Width, Min Feret, Max Feret 90), 3D characteristics (VolumeEqSphere, VolumeEqCylinder), and one EDF characteristic (EDFSurface). Factor 2 consists mainly of colour characteristics. The colour characteristics consist of (Mean Intensity, Mean Red, Mean Green, Mean Blue, Mean Brightness, Mean Density) and one EDF characteristic (EDF-Z).

For DF, another EDF characteristic (EDF Roughness) contributes to Factor 1 (**S3 Table**). In addition to colour characteristics identical to BF, (Hue Typical, Hue Variation, Mean Saturation, Bright Variation, Density Variation) contribute to Factor 2 as well. In contrast, Mean Red has a lower impact on this factor in DF. Recalculated morphological parameters (Convexity, Circularity, Roughness) also have a significant share in Factor 2. For Ph, the characteristics of Factor 1 (**S3 Table**) are identical to BF except for the EDF characteristic (EDF Surface) which has less weight. In addition to the colour characteristics identical to BF, the calculated colour characteristics (Intensity Variation, Hue Variation, Mean Saturation, Bright Variation) also contribute to Factor 2.

The significance of most of the measured morphological characteristics in the first factor confirms the suitability of determining pollen grains using size and shape characteristics. These characteristics are used both, in the manual evaluation of pollen grains [68,69] as well as in the evaluation of pollen grains by image processing.

Although morphological characteristics significantly contribute to Factor 1, they are not sufficient to discriminate the assessed taxa.

The colour characteristics of pollen grains play a significant role in F2 and also contribute to F4 and F5 in the case of BF. For DF and Ph, colour characteristics contribute significantly to F2 as well as to other factors to a lesser extent (**S3 Table**).

However, the first two factors are not sufficient for the accurate classification of pollen grains. In addition to the already mentioned colour characteristics, shape characteristics also contribute to other factors in BF. The shape characteristics involved in F3 are Circularity, Elongation, Shape Factor, Convexity, and Roughness. Other factors F4-F7 include both, recalculated colour values (Intensity Variation, Bright Variation, Density Variation, Hue Typical, Hue Variation) and EDF Roughness. For the DF microscopic technique, Elongation, Shape Factor, and Mean Red are mainly involved in F3. EDF-Z also contributes to other F4-F6 factors. In the Ph microscopic technique, Circularity, Shape Factor, Convexity, Roughness, and EDF-Z are involved in F3. Other factors of F4-F7 are formed by Density Variation, Elongation, Hue Typical, EDF Surface, and EDF Roughness.

### Multi-dimensional discriminant analysis

The microscopic methods were contrasted based on the correct classification rate (CCR) of the discriminant analysis (DA).

**Fig 5:**
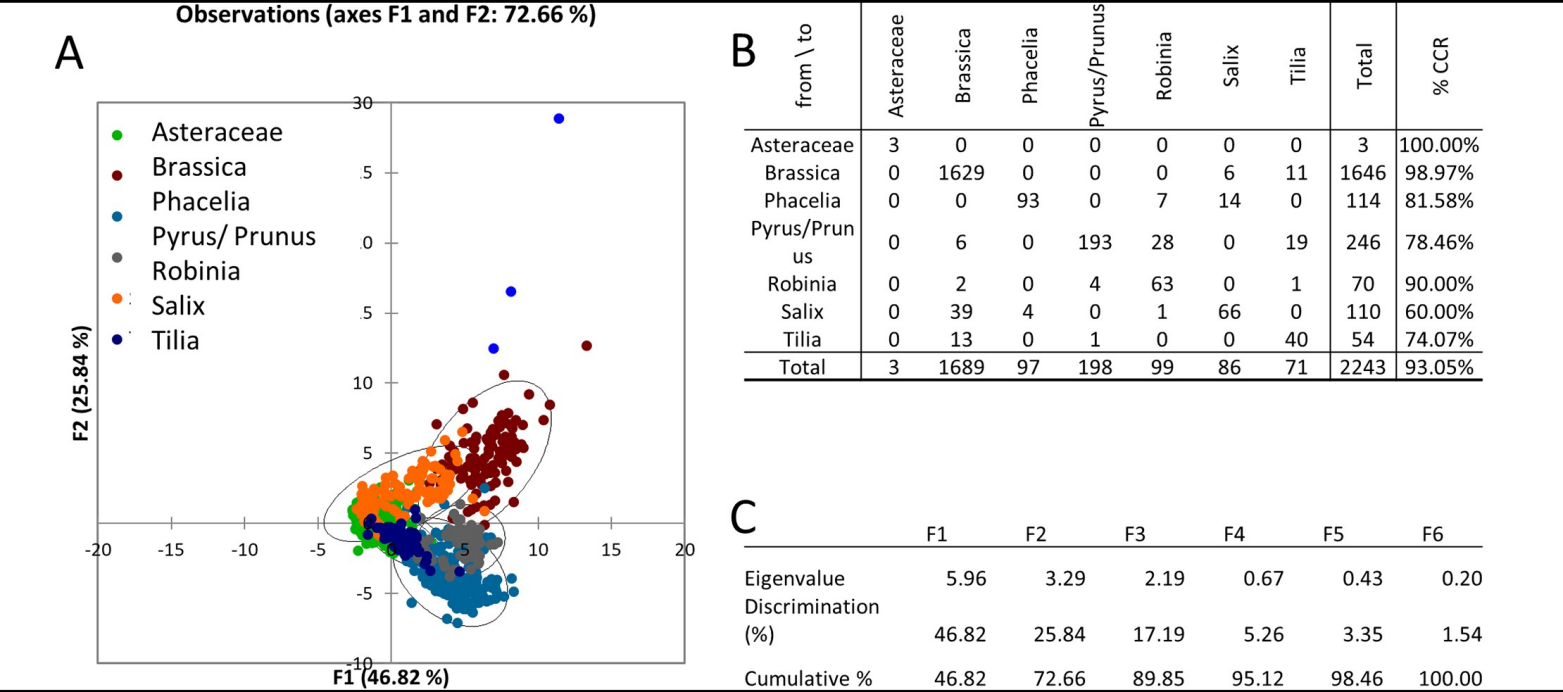
Ph discrimination analysis (A), confusion matrix for the estimated taxon (B), and eigenvalues table (C).

**Fig 6:**
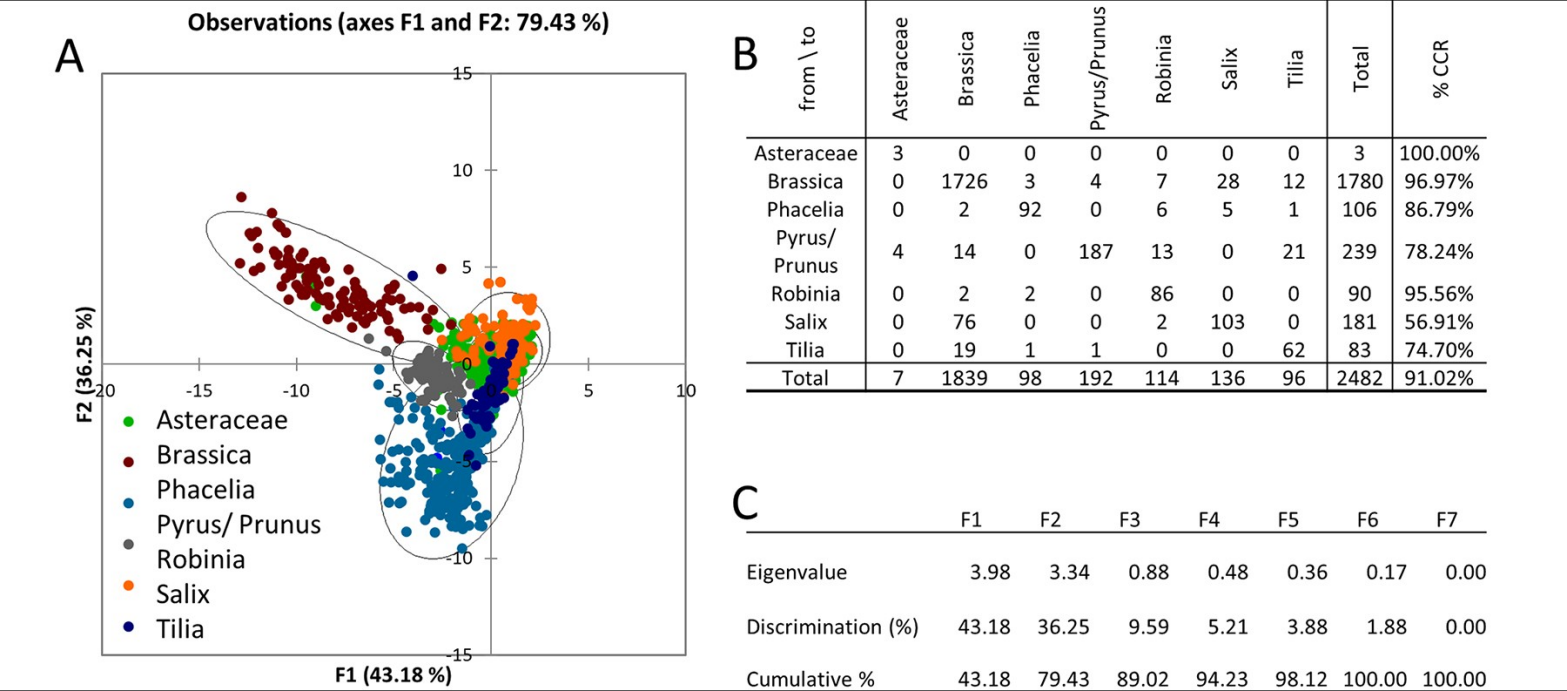
DF analysis (A), confusion matrix for the estimated taxon (B), and eigenvalues table (C).

The overall CCR of pollen grain discrimination based on the created model differed between the microscopic techniques used; the Ph reached 93.05%, DF 91.02%, and BF 88.88% (Figs 4–6).

For the Ph microscopic technique, the lowest CCR was achieved for the *Salix* taxon (60%) (Fig 5). We assume that the reason is a similar size and similar reticulate exine (Table 2, Fig 3) to *Brassica* and thus the classification into this taxon occurs incorrectly. Likewise, for the same reasons obviously, *Tilia* is incorrectly classified into the *Brassica* taxon with CCR 74.07%. Incorrect classification also occurs in *Pyrus/Prunus* 78.46% and *Robinia* 90%, where we assume that the reason is mainly the similar tricolpate shape of the pollen grain, see Fig 2. The use of Ph imaging technique thus proves to be the most suitable method for discriminating the studied pollen taxa (Fig 5). Ph is used in melissopalynological analysis as one of the microscopic methods [8,41] that allows 3D imaging of unstained structures. It can also be successfully used, for example, to identify honeydew elements [70]. In our study, its applicability for the semi-automated image processing method was also confirmed.

For DF microscopy, the least accurate discrimination was for the *Salix* taxon (56.91%), similar to Ph (Fig 6). Most misclassified pollens were classified as the *Brassica* taxon. Lower CCR was also for *Tilia* (74.70%) when most incorrectly classified pollen grains were assigned to the *Brassica* taxon. The pollen surface of these taxa forms a reticulate exine and discrimination using textural parameters is therefore difficult. CCR for *Pyrus/prunus* was 78.24% due to incorrect classification of part of the pollen into the *Brassica* and *Robinia* taxa. We attribute this incorrect discrimination to the tricolpate shape similar to *Robinia* and to the size similar to *Pyrus/prunus* and *Brasica* sp. Another factor of incorrect identification is also attributed to the shape similar to *Brassica* in some projections that are given by the polar or equatorial view. That is typical for heteropolar pollens.

The DF technique of automatic as well as semi-automatic analysis of pollen grains has been validated by several authors for pollen discrimination. In the study by Sevillano et al. [36], an automatic system for acquisition and identifying pollen grains was researched and the CCR of the DF method reached 97.86%. Lagerstrom et al. [24] reached 81.2% CCR when using DF. The difference in CCR values are caused not only by the different classification systems used but also by the different number of identified images and the number of classified pollen taxa.

**Fig 7:**
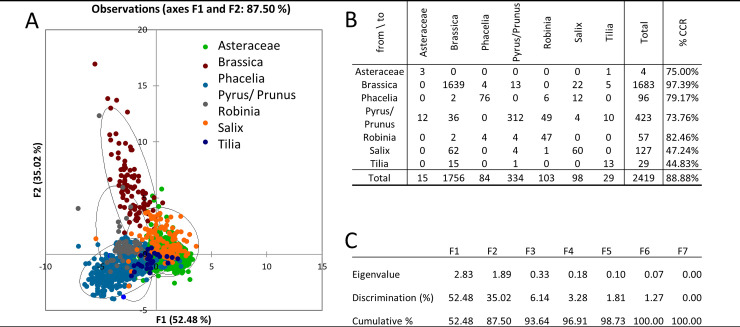
BF analysis (A), confusion matrix for the estimated taxon (B), and eigenvalues table (C).

For the BF technique, the lowest CCR was reached for *Tilia* (44.83%) and pollen grains that were most often incorrectly classified as *Brassica*. Even in this case, we attribute this to the heteropolarity of the *Tilia* pollen grain. Similar to Ph and DF, *Salix* was partially incorrectly classified into *Brassica* (Fig 7). With this technique, the CCR was below 50% (47.24%). BF is the most commonly used technique for evaluating pollen by both, visual inspection [1,39] and automatic analysis [18,24,71–73]. Differences in CCR vary between authors and range from 64% to 100% [6,63].

The lowest CCR for all microscopic methods was for Salix and Tillia taxa. Given the diversity of pollen taxa in honey, it is likely that even if automated analyses of pollen grains are established, precise identification of some taxa will be necessary. Either imaging-based methods such as SEM [21,74] or transmission electron microscopy [75] may be considered most appropriate. However, not all taxa can be distinguished from each other by melissopalynology methods [76] or by chemical markers [77]. However, specific identification of pollen taxa is also possible by methods based on other principles. Methods based on DNA detection can be considered as promising. When the DNA sequence is compared instead of the pollen grain morphology. For example, the chloroplast trnL barcoding fragment [78,79], rbcL DNA [80,81]. However, DNA methods also have their disadvantages which lead to a reduction in discriminatory power [82,83]. DNA methods allow for the examination of a larger number of pollen grains [80], on the other hand, the establishment of automated analysis will allow for a comparable number of grains to be examined and therefore a more accurate result.

Our study confirmed that discrimination of 7 botanical taxa in all microscopic methods using a membrane filter is possible. In contrast to paleoclimatology, for which this application is problematic [20], this quantitative method can be also used for automatic discrimination of pollen grains in melissopalynology. The discriminant model was created from 2243, 2419, and 2482 images of pollen grains for Ph, DF, and BF, respectively. The difference in the number of pollen grains is due to the different ability of the system to automatically focus and recognize the pollen grain. Conventional microscopic image acquisition systems are optimized for BF or fluorescence. Thus, most pollen grains were detected in BF; on the contrary, detection of some pollen grains was problematic for the Ph system and the number of detected and subsequently analysed pollen grains was therefore the lowest. In the case of routine capturing, the detection and focusing mechanism can be adjusted for Ph conditions. The BF microscopic method achieved the lowest CCR, however, it is the most commonly used method in the literature, for which there is a number of accompanying materials that facilitate the identification of pollen grains, especially for the human evaluator. Improvement of CCR in all compared techniques can be expected with the introduction of other forms of classification techniques, such as artificial neural networks [4] or convolutional neural networks [36].

## Conclusions

Various pollen descriptors were introduced in this study. The differences between pollen taxa were confirmed for microscopic methods used. Morphometric, volumetric, and colorimetric descriptors were mostly relevant for the discrimination with diverse variability for microscopic methods. 3D descriptors classified pollen taxa mostly in dark field, colour descriptors mostly in phase contrast. Likewise, EDF descriptors were helpful for the classification mostly in the dark field microscopy. The EDF Surface classified the most pollens into separate groups, compared to EDF Roughness and EDF-Z. None of one-dimensional descriptors can discriminate all pollen taxa into separate groups. For this reason, the multidimensional analysis was used. Discrimination analyses show different CCR. All microscopic methods can be used to classify pollen in honey into the appropriate taxa. The best technique for classification was phase contrast microscopy, where the CCR values achieved 93.05%. For dark field microscopy CCR value was 91.02%, and for bright field microscopy CCR reached 88.88%. However, although the method achieved a high CCR for the selected taxa, a large number of new taxa can be expected in the honey that will require human evaluation of pollen morphology for their correct identification. Our results provide background for future research where the taxa dataset will be increased, and new representative descriptors will be studied for pollen classification in honey.

## Supporting information

S1 Table Morphological descriptors 2D. (DOCX)

S2 Table Colour descriptors. (DOCX)

S3 Table Factor variable correlation matrix. (DOCX)

## References

[1] von der Ohe W, Persano Oddo L, Piana ML, Morlot M, Martin P. Harmonized methods of melissopalynology. Apidologie. 2004;35: S18–S25. doi: 10.1051/apido:2004050

[2] Shafiee S, Minaei S, Moghaddam-Charkari N, Ghasemi-Varnamkhasti M, Barzegar M. Potential application of machine vision to honey characterization. Trends in Food Science and Technology. 2013. pp. 174–177. doi: 10.1016/j.tifs.2012.12.004

[3] Feás X, Pires J, Iglesias A, Estevinho ML. Characterization of artisanal honey produced on the Northwest of Portugal by melissopalynological and physico-chemical data. Food Chem Toxicol. 2010;48: 3462–3470. doi: 10.1016/j.fct.2010.09.024 20870005

[4] del Pozo-Baños M, Ticay-Rivas JR, Alonso JB, Travieso CM. Features extraction techniques for pollen grain classification. Neurocomputing. 2015;150: 377–391. doi: 10.1016/j.neucom.2014.05.085

[5] Pospiech M, Javůrková Z, Tremlová B, Běhalová H. Characterization of fruit trees pollen. Potravin Slovak J Food Sci. 2019;13: 634–643. doi: 10.5219/1096

[6] LI P, Treloar WJ, Flenley JR, Empson L. Towards automation of palynology 2: the use of texture measures and neural network analysis for automated identification of optical images of pollen grains. J Quat Sci. 2004;19: 755–762. doi: 10.1002/jqs.874

[7] Li P, Flenley JR. Pollen texture identification using neural networks. Grana. 1999;38: 59–64. doi: 10.1080/001731300750044717

[8] Jones GD, Bryant VM. Pollen Studies of East Texas Honey. Palynology. 2014;38: 242–258. doi: 10.1080/01916122.2014.899276

[9] Soares EL, Landi LADC, Gasparino EC. Additions to the knowledge of the pollen morphology of some Fabaceae from the cerrado’s forest patches of Brazil. Palynology. 2020; 1–16. doi: 10.1080/01916122.2020.1804007

[10] Piper T, Piper J. Universal variable brightfield-darkfield contrast: A variant technique for improved imaging of problematic specimens in light microscopy. Microscopy and Microanalysis. 2013. pp. 1092–1105. doi: 10.1017/S143192761300158X 23702156

[11] Determann H, Lepusch F. Darkfield microscopy, phase contrast microscopy, interference contrast microscopy. Microsc Its Appl. 1981; 18–24. Available: https://scholar.google.cz/scholar?hl=cs&as_sdt=0%2C5&q=Darkfield+microscopy%2C+phase+contrast+microscopy%2C+interference+contrast+microscopy&btnG.

[12] Piper T, Piper J. Variable phase bright-field contrast—An alternative illumination technique for improved imaging in transparent specimens. Microsc Microanal. 2013;19: 11–21. doi: 10.1017/S1431927612013323 23237494

[13] Yin Z, Kanade T, Chen M. Understanding the phase contrast optics to restore artifact-free microscopy images for segmentation. Med Image Anal. 2012;16: 1047–1062. doi: 10.1016/j.media.2011.12.006 22386070 PMC3372640

[14] Ullah F, Nasar Shah S, Zaman W, Zafar M, Ahmad M, Ayaz A, et al. Using palynomorphological characteristics for the identification of species of Alsinoideae (Caryophyllaceae): a systematic approach. 2019 [cited 13 Jul 2021]. doi: 10.1080/00173134.2019.1569719

[15] Gul S, Ahmad M, Zafar M, Bahadur S, Zaman W, Ayaz A, et al. Palynological characteristics of selected Lamioideae taxa and its taxonomic significance. Microsc Res Tech. 2021;84: 471–479. doi: 10.1002/jemt.23603 32959483

[16] Bahadur S, Ahmad M, Zafar M, Sultana S, Begum N, Ashfaq S, et al. Palyno-anatomical studies of monocot taxa and its taxonomic implications using light and scanning electron microscopy. Microsc Res Tech. 2019;82: 373–393. doi: 10.1002/jemt.23179 30575189

[17] Zhang M, Zhang K, Feng Q, Wang J, Kong J, Lu Y. A novel image retrieval method based on hybrid information descriptors. J Vis Commun Image Represent. 2014;25: 1574–1587. doi: 10.1016/j.jvcir.2014.06.016

[18] Kaya Y, Erez ME, Karabacak O, Kayci L, Fidan M. An automatic identification method for the comparison of plant and honey pollen based on GLCM texture features and artificial neural network. Grana. 2013;52: 71–77. doi: 10.1080/00173134.2012.754050

[19] Redondo R, Bueno G, Chung F, Nava R, Víctor Marcos J, Cristóbal G, et al. Pollen segmentation and feature evaluation for automatic classification in bright-field microscopy. Comput Electron Agric. 2015;110: 56–69. doi: 10.1016/j.compag.2014.09.020

[20] Stillman EC, Flenleyt JR. THE NEEDS AND PROSPECTS FOR AUTOMATION IN PALYNOLOGY. Quat Sci Rev. 1996.

[21] Treloar WJ, Taylor GE, Flenley JR. Towards automation of palynology 1: analysis of pollen shape and ornamentation using simple geometric measures, derived from scanning electron microscope images. J Quat Sci. 2004;19: 745–754. doi: 10.1002/jqs.871

[22] Zhang Y, Fountain DW, Hodgson RM, Flenley JR, Gunetileke S. Towards automation of palynology 3: pollen pattern recognition using Gabor transforms and digital moments. J Quat Sci. 2004;19: 763–768. doi: 10.1002/jqs.875

[23] Holt K, Allen G, Hodgson R, Marsland S, Flenley J. Progress towards an automated trainable pollen location and classifier system for use in the palynology laboratory. Rev Palaeobot Palynol. 2011;167: 175–183. doi: 10.1016/j.revpalbo.2011.08.006

[24] Lagerstrom R, Holt K, Arzhaeva Y, Bischof L, Haberle S, Hopf F, et al. Pollen image classification using the classifynder system: Algorithm comparison and a case study on New Zealand honey. Adv Exp Med Biol. 2015;823: 207–226. doi: 10.1007/978-3-319-10984-8_12 25381110

[25] Punyasena SW, Tcheng DK, Wesseln C, Mueller PG. Classifying black and white spruce pollen using layered machine learning. New Phytol. 2012;196: 937–944. doi: 10.1111/j.1469-8137.2012.04291.x 22943455

[26] Jacinto-Pimienta SY, Mendoza-Hernández JHR, Zaldivar-Cruz JM, Sol-Sánchez Á, Vargas-Villamil LM, Reyes-Sánchez CA. El uso de componentes principales en la clasificación melisopalinológica de la miel de Apis mellifera L.* Use of principal component in melissopalynology classification of honey fromApis mellifera L. Rev Mex Ciencias Agrícolas. 2016;14: 2831–2840.

[27] Pospiech M, Ljasovská S, Titěra D, Kružík V, Javurková Z, Tremlová B. Pollen Diversity in Honey of the Czech Republic in the 2019 Season. Potravin Slovak J Food Sci. 2020;14: 1115–1123. doi: 10.5219/1504

[28] Pospiech M, Javůrková Z, Hrabec P, Čížková H, Titěra D, Štarha P, et al. Physico-Chemical and Melissopalynological Characterization of Czech Honey. Appl Sci. 2021;11: 4989. doi: 10.3390/app11114989

[29] Chica M. Authentication of bee pollen grains in bright-field microscopy by combining one-class classification techniques and image processing. Microsc Res Tech. 2012. doi: 10.1002/jemt.22091 22736501

[30] Martõ Ânez AM, Kak AC. PCA versus LDA.

[31] Wu Y, Wang C. Extended depth of focus image for phytolith analysis. J Archaeol Sci. 2009;36: 2253–2257. doi: 10.1016/j.jas.2009.06.010

[32] Ticay-Rivas JR, Del Pozo-Baños M, Travieso CM, Arroyo-Hernández J, Pérez ST, Alonso JB, et al. Pollen classification based on geometrical, descriptors and colour features using decorrelation stretching method. IFIP Advances in Information and Communication Technology. Springer New York LLC; 2011. pp. 342–349. doi: 10.1007/978-3-642-23960-1_41

[33] Naila A, Flint SH, Sulaiman AZ, Ajit A, Weeds Z. Classical and novel approaches to the analysis of honey and detection of adulterants. Food Control. Elsevier Ltd; 2018. pp. 152–165. doi: 10.1016/j.foodcont.2018.02.027

[34] Erdtman G. ON POLLEN AND SPORE TERMINOLOGY.

[35] Punt W, Hoen PP, Blackmore S, Nilsson S, Le Thomas A. Glossary of pollen and spore terminology. Rev Palaeobot Palynol. 2007;143: 1–81. doi: 10.1016/j.revpalbo.2006.06.008

[36] Sevillano V, Holt K, Aznarte JL. Precise automatic classification of 46 different pollen types with convolutional neural networks. PLoS One. 2020;15: 1–15. doi: 10.1371/journal.pone.0229751 32574174 PMC7310700

[37] El-Labban M. Beekeepers’ Guide For Pollen Identification Of Honey. Lebanon: Mohammad El-Labban; 2020.

[38] von der Ohe K, Von Der Ohe W. Celle’s melissopalynological collection. 3. edition. Celle: LAVES—institut fur Bienenkunde Celle; 2007.

[39] Kale Sniderman JM, Matley KA, Haberle SG, Cantrill DJ. Pollen analysis of Australian honey. PLoS One. 2018;13: 1–24. doi: 10.1371/journal.pone.0197545 29768495 PMC5955576

[40] Boucher A, Hidalgo PJ, Thonnat M, Belmonte J, Galan C, Bonton P, et al. Development of a semi-automatic system for pollen recognition. Aerobiologia. Springer; 2002. pp. 195–201. doi: 10.1023/A:1021322813565

[41] Kaur H, Sharma SK, Sharma PC. Palynological Study of Pollens of Some Important Bee Floral Plants in Kangra District of Himachal Pradesh. Himachal J Agric Res. 2019 Dec. Available: http://hjar.in/index.php/hjar/article/view/150382.

[42] Radice S, Ontivero M, Andorno A, Dessy S. Floral morphology and pollen viability of the “forastero” cultivar [prunus persica (l.) batsch], as modified by the rootstock. Acta Horticulturae. 2004. doi: 10.17660/ActaHortic.2004.658.8

[43] von der Ohe K, Dustmann JH. Scanning electron microscopic studies of pollen from honey. III. The harmomegathy mechanism and its effect on the exine structure of different pollen types. In German. Apidologie. 1990;21: 293–302. doi: 10.1051/apido:19900404

[44] Lozano-Vega G, Benezeth Y, Marzani F, Boochs F. Analysis of relevant features for pollen classification. IFIP Advances in Information and Communication Technology. 2014. pp. 395–404. doi: 10.1007/978-3-662-44654-6_39

[45] Kopanja L, Lončar B, Žunić D, Tadić M. Nanoparticle shapes: Quantification by elongation, convexity and circularity measures. J Electr Eng. 2019;70: 44–50. doi: 10.2478/jee-2019-0040

[46] Halbritter H, Ulrich S, Grímsson F, Weber M, Zetter R, Hesse M, et al. Illustrated Pollen Terminology. Illustrated Pollen Terminology. 2018. doi: 10.1007/978-3-319-71365-6

[47] Pedersen B, Bailey DG, Hodgson RM, Holt K, Marsland S. Model and feature selection for the classification of dark field pollen images using the classifynder system. International Conference Image and Vision Computing New Zealand. IEEE Computer Society; 2018. pp. 1–5. doi: 10.1109/IVCNZ.2017.8402498

[48] Cootes TF, Cooper DH, Taylor CJ, Graham J. Trainable method of parametric shape description. Image Vis Comput. 1992;10: 289–294. doi: 10.1016/0262-8856(92)90044-4

[49] Sun S, Sundararaghavan V. A probabilistic crystal plasticity model for modeling grain shape effects based on slip geometry. Acta Mater. 2012;60: 5233–5244. doi: 10.1016/j.actamat.2012.05.039

[50] Latypov MI, Kühbach M, Beyerlein IJ, Stinville JC, Toth LS, Pollock TM, et al. Application of chord length distributions and principal component analysis for quantification and representation of diverse polycrystalline microstructures. Mater Charact. 2018;145: 671–685. doi: 10.1016/j.matchar.2018.09.020

[51] Holt KA, Bebbington MS. Separating Morphologically Similar Pollen Types Using Basic Shape Features from Digital Images: A Preliminary Study. Appl Plant Sci. 2014;2: 1400032. doi: 10.3732/apps.1400032 25202650 PMC4141716

[52] Agimelen OS, Hamilton P, Haley I, Nordon A, Vasile M, Sefcik J, et al. Estimation of particle size distribution and aspect ratio of non-spherical particles from chord length distribution. Chem Eng Sci. 2015;123: 629–640. doi: 10.1016/j.ces.2014.11.014

[53] Bonton P, Boucher A, Thonnat M, Tomczak R, Hidalgo PJ, Belmonte J, et al. Colour Image in 2D and 3D Microscopy for the Automation of Pollen Rate Measurement. Image Anal Stereol. 2004;21: 25–30. doi: 10.5566/ias.v21.p25-30

[54] Gallardo-Caballero R, García-Orellana CJ, García-Manso A, González-Velasco HM, Tormo-Molina R, Macías-Macías M. Precise pollen grain detection in bright field microscopy using deep learning techniques. Sensors (Switzerland). 2019;19: 3583. doi: 10.3390/s19163583 31426511 PMC6720915

[55] Bagheri GH, Bonadonna C, Manzella I, Vonlanthen P. On the characterization of size and shape of irregular particles. Powder Technol. 2015;270: 141–153. doi: 10.1016/j.powtec.2014.10.015

[56] Doyle JA. Early evolution of angiosperm pollen as inferred from molecular and morphological phylogenetic analyses. Grana. Taylor and Francis A.S.; 2005. pp. 227–251. doi: 10.1080/00173130500424557

[57] Hebda RJ, Chinnappa CC, Smith BM. Pollen morphology of the rosaceae of Western Canada:I. Agrimonia to crataegus. Grana. 1988;27: 95–113. doi: 10.1080/00173138809432836

[58] Marcos JV, Nava R, Cristóbal G, Redondo R, Escalante-Ramírez B, Bueno G, et al. Automated pollen identification using microscopic imaging and texture analysis. Micron. 2015;68: 36–46. doi: 10.1016/j.micron.2014.09.002 25259684

[59] Mills M, Bonetti J, Brettell T, Quarino L. Differentiation of human hair by colour and diameter using light microscopy, digital imaging and statistical analysis. J Microsc. 2018;270: 27–40. doi: 10.1111/jmi.12646 28960300

[60] Holt KA, Bennett KD. Principles and methods for automated palynology. New Phytol. 2014;203: 735–742. doi: 10.1111/nph.12848 25180326

[61] Clarke EL, Treanor D. Colour in digital pathology: A review. Histopathology. Blackwell Publishing Ltd; 2017. pp. 153–163. doi: 10.1111/his.13079 27607349

[62] Deagle RC, Wee TL (Erika), Brown CM. Reproducibility in light microscopy: Maintenance, standards and SOPs. Int J Biochem Cell Biol. 2017;89: 120–124. doi: 10.1016/j.biocel.2017.06.008 28606390

[63] Gonçalves AB, Souza JS, Silva GG da, Cereda MP, Pott A, Naka MH, et al. Feature extraction and machine learning for the classification of Brazilian Savannah pollen grains. Kestler HA, editor. PLoS One. 2016;11: e0157044. doi: 10.1371/journal.pone.0157044 27276196 PMC4898734

[64] Mitsumoto K, Yabusaki K, Aoyagi H. Classification of pollen species using autofluorescence image analysis. J Biosci Bioeng. 2009;107: 90–94. doi: 10.1016/j.jbiosc.2008.10.001 19147117

[65] Stacherzak A, Hájek L, Hełdak M. Changes in the use of agricultural land in Poland and Czech Republic. J Ecol Eng. 2019;20: 211–221. doi: 10.12911/22998993/109869

[66] Čeksteryte V, Kurtinaitiene B, Balžekas J. Pollen diversity in honey collected from Lithuania’s protected landscape areas. Proc Est Acad Sci. 2013;62: 277–282. doi: 10.3176/proc.2013.4.08

[67] Canini A, Pichichero E, Alesian D, Canuti L, Leonardi D. Nutritional and botanical interest of honey collected from protected natural areas. Plant Biosyst. 2009;143: 62–70. doi: 10.1080/11263500802633543

[68] Ullah F, Zafar M, Ahmad M, Dilbar S, Shah SN, Sohail A, et al. Pollen morphology of subfamily Caryophylloideae (Caryophyllaceae) and its taxonomic significance. Verkade P, editor. Microsc Res Tech. 2018;81: 704–715. doi: 10.1002/jemt.23026 29582513

[69] Al-Watban AA, Al-Mogren E, Doaigey AR, Zaidy M El. African Journal of Plant Science Pollen morphology of seven wild species of Acacia in Saudi Arabia. African J Plant Sci. 2013;7: 602–607. doi: 10.5897/AJPS2012.0989

[70] Mura-Mészáros A, Magyar D. Fungal Honeydew Elements as Potential Indicators of the Botanical and Geographical Origin of Honeys. Food Anal Methods. 2017;10: 3079–3087. doi: 10.1007/s12161-017-0862-x

[71] Daood A, Ribeiro E, Bush M. Pollen grain recognition using deep learning. Lecture Notes in Computer Science (including subseries Lecture Notes in Artificial Intelligence and Lecture Notes in Bioinformatics). Springer Verlag; 2016. pp. 321–330. doi: 10.1007/978-3-319-50835-1_30

[72] Sevillano V, Aznarte JL. Improving classification of pollen grain images of the POLEN23E dataset through three different applications of deep learning convolutional neural networks. Rutherford S, editor. PLoS One. 2018;13: e0201807. doi: 10.1371/journal.pone.0201807 30216353 PMC6138340

[73] Daood A, Ribeiro E, Bush M. Sequential recognition of pollen grain Z-stacks by combining CNN and RNN. Proceedings of the 31st International Florida Artificial Intelligence Research Society Conference, FLAIRS 2018. 2018.

[74] Saklani S, V. K M. Scanning Electron Microscopic Study on Pollens of 8 Bee Floral Resources from Kangra Hills, Himachal Pradesh, India. Int J Biotech Trends Technol. 2020;10: 67–71. doi: 10.14445/22490183/ijbtt-v10i1p611

[75] Ajipe JO, Adebayo MB. The Significance of Palynology in Socio-economic Development in Nigeria. Adv Multidiscip Sci Res J. 2018;4: 51–58. Available: https://www.researchgate.net/publication/325257228.

[76] Prosser SWJ, Hebert PDN. Rapid identification of the botanical and entomological sources of honey using DNA metabarcoding. Food Chem. 2017;214: 183–191. doi: 10.1016/j.foodchem.2016.07.077 27507464

[77] Kaškoniene V, Venskutonis PR, Kaškonienė V, Venskutonis PR. Floral Markers in Honey of Various Botanical and Geographic Origins: A Review. Compr Rev Food Sci Food Saf. 2010;9: 620–634. doi: 10.1111/j.1541-4337.2010.00130.x 33467823

[78] Chiara B, Francesco C, Fulvio B, Paola M, Annalisa G, Stefania S, et al. Exploring the botanical composition of polyfloral and monofloral honeys through DNA metabarcoding. Food Control. 2021;128: 108175. doi: 10.1016/J.FOODCONT.2021.108175

[79] Utzeri VJ, Ribani A, Schiavo G, Bertolini F, Bovo S, Fontanesi L. Application of next generation semiconductor based sequencing to detect the botanical composition of monofloral, polyfloral and honeydew honey. Food Control. 2018;86: 342–349. doi: 10.1016/j.foodcont.2017.11.033

[80] Hawkins J, De Vere N, Griffith A, Ford CR, Allainguillaume J, Hegarty MJ, et al. Using DNA metabarcoding to identify the floral composition of honey: A new tool for investigating honey bee foraging preferences. PLoS One. 2015;10. doi: 10.1371/journal.pone.0134735 26308362 PMC4550469

[81] Galimberti A, De Mattia F, Bruni I, Scaccabarozzi D, Sandionigi A, Barbuto M, et al. A DNA barcoding approach to characterize pollen collected by honeybees. PLoS One. 2014;9. doi: 10.1371/journal.pone.0109363 25296114 PMC4190116

[82] Bell KL, Burgess KS, Okamoto KC, Aranda R, Brosi BJ. Review and future prospects for DNA barcoding methods in forensic palynology. Forensic Sci Int Genet. 2016;21: 110–116. doi: 10.1016/j.fsigen.2015.12.010 26751251

[83] Hollingsworth PM, Graham SW, Little DP. Choosing and Using a Plant DNA Barcode. doi: 10.1371/journal.pone.0019254 21637336 PMC3102656

