# Supplementary material for: Identification of pollen taxa by different microscopy techniques
Source: PLoS One. 2021 Sep 1;16(9):e0256808. doi: 10.1371/journal.pone.0256808 (PMC8409677; doi:10.1371/journal.pone.0256808)
Supplement: S1 Table — (DOCX) [file pone.0256808.s001.docx]

**S1 Table. Morphological descriptors 2D**

| **Area** | Area is a principal size criterion. In a non-calibrated system, it expresses the number of pixels; in a calibrated one, it expresses the real area. | $Area=\sum_{\forall Pixel \in Object} 1$ |
| --- | --- | --- |
| **EqDiameter** (Equivalent Diameter) | A size feature derived from the area. It determines the diameter of a circle with the same area as the measured object: | $Eqdiameter2D=\sqrt{\frac{4*Area}{\pi}}$ |
| **Perimeter** **Contour** | Total boundary length computed from object contours. Compared to Perimeter, it is more accurate (especially on elongated objects) but slower to get the result. | $Perimeter=\sum_{\forall Pixel \in Contour} 1$ |
| **Mean Chord** | MeanChord is the mean value of secants in the 0, 45, 90 and 135 degrees directions. It is a derived feature and is calculated from the Area and mean projection according to the following formula. | $MeanChord=\frac{4*Area}{\left( {Pr}_{0}+{Pr}_{45}+{Pr}_{90}+{Pr}_{135} \right)}$ |
| **Lenght** | Length is a derived feature appropriate for elongated or thin structures. As based on the rod model, it is useful for calculating length of medial axis of thin rods. | $Length =\frac{Perimetr+\sqrt{\left( {Perimetr}^{2}-16*Area \right)}}{4}$ |
| **Width** | Width is a derived feature appropriate for elongated or thin structures. It is based on the rod model and is calculated according to the following formula: | $Width=\frac{Area}{Lenght}$ |
| **MinFeret** | The MinFeret value is the minimal value of the set of Feret's diameters. Generally (for convex objects), Feret's diameter at angle α equals the projected length of object at angle α , α (0,180); NIS-Elements AR calculates Feret's diameter for α =0,10,20, 30, ..., 180. | $MinFeret=\min_{\forall\alpha\in\left\langle0,180 \right\rangle} Feret\left( \alpha\right)$ |
| **MaxFeret90** | The MaxFeret is the maximal value of the set of Feret's diameters. Generally (for convex objects), Feret's diameter at angle α equals the projected length of object at angle α , α (0,180); NIS-Elements AR calculates Feret's diameter for α =0, 10, 20, 30, ..., 180.  The MaxFeret90 is a length projected across the MaxFeret diameter. | $MaxFeret=\max_{\forall\alpha\in\left\langle0,180 \right\rangle} Feret\left( \alpha\right)$ |
| **Circularity** | Circularity equals to 1 only for circles; all other shapes are characterized by circularity smaller than 1. It is a derived shape measure, calculated from the area and perimeter. This feature is useful for examining shape characteristics. | $Circularity=\frac{4*\pi*Area}{{Perimetr}^{2}}$ |
| **Elongation** | Characterizes the object shape. Ratio of MaxFeret and MinFeret features. | $Elongation= \frac{MaxFeret}{MinFeret}$ |
| **Shape** **Factor** | This parameter is to define whether the object is rough or not. | $ShapeFactor=\frac{4*\pi*Area}{{Conver hull perimeter}^{2}}$ |
| **Convexity** | Indicates convexity of the object edges. | $Convexity= \frac{Area}{Convex Hull Area}$ |
