# Supplementary material for: Identification of pollen taxa by different microscopy techniques
Source: PLoS One. 2021 Sep 1;16(9):e0256808. doi: 10.1371/journal.pone.0256808 (PMC8409677; doi:10.1371/journal.pone.0256808)
Supplement: S2 Table — (DOCX) [file pone.0256808.s002.docx]

**S2 Table. Colour descriptors**

| **Mean Intensity** | MeanIntensity value is derived from the intensity histogram. It is the arithmetic mean of pixel intensities. | $I=\frac{1}{3} \left( R,G,B \right)$  $Mean I=\frac{1}{n}\sum_{n} I_{i}$ |
| --- | --- | --- |
| **Intensity Variation** | IntensityVariation is derived from an intensity histogram. It is an usual standard deviation of intensity values. This feature describes the inner structure of an object or a field. | $Var I=\frac{1}{n}\sum_{n} {(I_{i} - Mean I )}^{2}$ |
| **Mean Red** | Arithmetic mean of pixel intensities of one image component. | $Mean R=\frac{1}{n}\sum_{n} R_{i}$ |
| **Mean Green** | Arithmetic mean of pixel intensities of one image component. | $Mean G=\frac{1}{n}\sum_{n} G_{i}$ |
| **Mean Blue** | Arithmetic mean of pixel intensities of one image component. | $Mean B=\frac{1}{n}\sum_{n} B_{i}$ |
| **Hue Typical** | HueTypical (H) is the hue value with maximum frequency in a hue value histogram. This feature describes the most frequent hue (color) in an object or field. | $\alpha=\left( 2R-G-B \right)$  $\beta=\sqrt{3}\left( G-B \right)$  $H=atan2\left( \beta,\alpha\right)$ |
| **Hue Variation** | HueVariation is the usual standard deviation of hue values. This feature describes hue (color) distribution of inner structure of an object or field. | $Var H=\frac{1}{n}\sum_{n} {(H_{i} - Mean H )}^{2}$ |
| **Mean Saturation** | Arithmetic mean of saturation values of pixels (S). | $I=\frac{1}{3} \left( R,G,B \right)$  $m=\mathrm{mi}n \left( R,G,B \right)$  $S^{I}=\left\{ \begin{aligned} 0, if I=0 \\ 1-\frac{m}{I}, \mathrm{otherwise} \end{aligned} \right.$  $Mean S^{I}=\frac{1}{n}\sum_{n} S_{i}^{I}$ |
| **Mean Brightness** | Arithmetic mean of brightness values of pixels. | $Br=0.299R+0.587G +0.114B$  $Mean Br=\frac{1}{n}\sum_{n} {Br}_{i}$ |
| **Bright Variation** | It is the usual standard deviation of brightness values. | $Var Br=\frac{1}{n}\sum_{n} \left( {BR}_{i}-MeanBR \right)^{2}$ |
| **Mean Density** | Arithmetic mean of density values of pixels. | $maxvalue = 255, 8bit image$ $maxvalue = 65535$, 16bit image  $D=\frac{R+G+B}{3 maxvalue}$  $Mean D=\frac{1}{n}\sum_{n} D_{i}$ |
| **Density Variation** | Density variation is derived from density values. It is the usual standard deviation of density values. | $Var D=\frac{1}{n}\sum_{n} {(D_{i} - Mean D )}^{2}$ |
