# Supplementary material for: Identification of pollen taxa by different microscopy techniques
Source: PLoS One. 2021 Sep 1;16(9):e0256808. doi: 10.1371/journal.pone.0256808 (PMC8409677; doi:10.1371/journal.pone.0256808)
Supplement: S3 Table — (DOCX) [file pone.0256808.s003.docx]

**S3 Table. Factor variable correlation matrix.**

| BF | F1 | F2 | F3 | F4 | F5 | F6 | F7 |
| --- | --- | --- | --- | --- | --- | --- | --- |
| Area [µm²] | **0,952** | 0,277 | 0,030 | 0,074 | 0,007 | 0,091 | 0,039 |
| EqDiameter [µm] | **0,943** | 0,298 | 0,066 | 0,082 | 0,016 | 0,056 | 0,038 |
| VolumeEqSphere [µm³] | **0,939** | 0,252 | 0,004 | 0,066 | 0,001 | 0,119 | 0,038 |
| VolumeEqCylinder [µm³] | **0,925** | 0,241 | 0,006 | 0,062 | -0,008 | 0,114 | 0,036 |
| PerimeterContour [µm] | **0,941** | 0,270 | -0,162 | 0,050 | 0,084 | -0,018 | 0,044 |
| MeanChord [µm] | **0,879** | 0,310 | 0,308 | 0,111 | -0,059 | 0,128 | 0,031 |
| Length [µm] | **0,851** | 0,290 | -0,220 | 0,048 | 0,074 | 0,161 | 0,035 |
| Width [µm] | **0,845** | 0,243 | 0,342 | 0,096 | -0,048 | -0,092 | 0,032 |
| MinFeret [µm] | **0,897** | 0,237 | 0,248 | 0,087 | 0,010 | -0,219 | 0,041 |
| MaxFeret90 [µm] | **0,897** | 0,219 | 0,242 | 0,075 | 0,020 | -0,225 | 0,038 |
| Circularity | -0,307 | 0,009 | **0,886** | 0,098 | -0,260 | 0,207 | -0,019 |
| Elongation | -0,042 | 0,074 | **-0,734** | -0,060 | 0,029 | 0,630 | 0,028 |
| ShapeFactor | -0,286 | -0,032 | **0,845** | 0,083 | -0,135 | -0,189 | -0,078 |
| Convexity | -0,330 | 0,062 | **0,657** | 0,092 | -0,241 | 0,402 | -0,024 |
| Roughness | -0,212 | 0,044 | **0,521** | 0,067 | -0,247 | 0,481 | 0,046 |
| MeanIntensity | 0,482 | **-0,870** | 0,039 | 0,081 | -0,014 | 0,027 | -0,046 |
| IntensityVariation | 0,432 | -0,310 | 0,173 | **-0,822** | 0,065 | 0,062 | 0,010 |
| MeanRed | 0,423 | **-0,846** | 0,226 | 0,065 | 0,151 | 0,011 | -0,023 |
| MeanGreen | 0,481 | **-0,864** | 0,040 | 0,086 | -0,003 | 0,033 | -0,053 |
| MeanBlue | 0,497 | **-0,796** | -0,198 | 0,086 | -0,228 | 0,035 | -0,056 |
| HueTypical | 0,123 | 0,120 | -0,354 | -0,182 | **-0,878** | -0,152 | 0,133 |
| HueVariation | 0,073 | -0,049 | 0,082 | -0,204 | **-0,565** | -0,184 | 0,176 |
| MeanSaturation | -0,231 | 0,206 | **0,599** | -0,071 | 0,510 | -0,031 | 0,035 |
| MeanBrightness | 0,482 | **-0,870** | 0,039 | 0,081 | -0,014 | 0,027 | -0,046 |
| BrightVariation | 0,431 | -0,310 | 0,173 | **-0,822** | 0,065 | 0,062 | 0,010 |
| MeanDensity | -0,463 | **0,870** | -0,014 | -0,129 | 0,033 | -0,023 | 0,056 |
| DensityVariation | 0,109 | 0,182 | 0,163 | **-0,887** | 0,090 | 0,030 | 0,067 |
| EdfSurface [µm²] | **0,893** | 0,348 | 0,009 | 0,002 | -0,060 | 0,057 | -0,274 |
| EdfRoughness | 0,022 | 0,279 | -0,067 | -0,190 | -0,172 | -0,058 | **-0,719** |
| Edf-Z [µm] | 0,020 | **0,361** | -0,106 | -0,052 | -0,133 | 0,056 | -0,185 |
| *Values in bold correspond for each variable to the factor for which the squared cosine is the largest* | | | | | | | |

| DF | F1 | F2 | F3 | F4 | F5 | F6 |
| --- | --- | --- | --- | --- | --- | --- |
| Area [µm²] | **0,828** | 0,545 | 0,049 | -0,038 | 0,073 | -0,044 |
| EqDiameter [µm] | **0,843** | 0,508 | 0,094 | -0,039 | 0,090 | -0,029 |
| VolumeEqSphere [µm³] | **0,798** | 0,561 | 0,012 | -0,034 | 0,055 | -0,055 |
| VolumeEqCylinder [µm³] | **0,797** | 0,538 | 0,011 | -0,035 | 0,051 | -0,039 |
| PerimeterContour [µm] | **0,780** | 0,617 | -0,061 | 0,035 | 0,004 | -0,006 |
| MeanChord [µm] | **0,863** | 0,351 | 0,262 | -0,119 | 0,180 | -0,050 |
| Length [µm] | 0,631 | **0,660** | -0,103 | 0,011 | 0,041 | -0,138 |
| Width [µm] | **0,873** | 0,237 | 0,259 | -0,081 | 0,109 | 0,100 |
| MinFeret [µm] | **0,885** | 0,359 | 0,200 | 0,006 | 0,009 | 0,173 |
| MaxFeret90 [µm] | **0,884** | 0,349 | 0,196 | 0,003 | 0,000 | 0,179 |
| Circularity | -0,028 | **-0,688** | 0,640 | -0,230 | 0,252 | 0,011 |
| Elongation | -0,404 | 0,402 | **-0,557** | -0,035 | 0,078 | -0,399 |
| ShapeFactor | 0,016 | -0,664 | **0,670** | -0,139 | 0,157 | 0,138 |
| Convexity | -0,288 | **-0,554** | 0,377 | -0,302 | 0,361 | -0,257 |
| Roughness | -0,177 | **-0,454** | 0,204 | -0,325 | 0,320 | -0,302 |
| MeanIntensity | -0,563 | **0,734** | 0,283 | -0,188 | -0,172 | 0,006 |
| IntensityVariation | 0,416 | **-0,630** | -0,229 | -0,457 | -0,275 | 0,221 |
| MeanRed | -0,389 | 0,457 | **0,584** | -0,063 | -0,474 | -0,132 |
| MeanGreen | -0,567 | **0,669** | 0,398 | -0,108 | -0,221 | -0,011 |
| MeanBlue | -0,549 | **0,784** | 0,018 | -0,266 | 0,038 | 0,082 |
| HueTypical | -0,500 | **0,530** | -0,451 | -0,292 | 0,267 | 0,046 |
| HueVariation | -0,376 | **0,519** | -0,438 | -0,456 | 0,193 | 0,132 |
| MeanSaturation | 0,444 | **-0,750** | 0,111 | 0,266 | -0,203 | -0,191 |
| MeanBrightness | -0,563 | **0,734** | 0,283 | -0,188 | -0,172 | 0,006 |
| BrightVariation | 0,416 | **-0,630** | -0,229 | -0,457 | -0,275 | 0,221 |
| MeanDensity | 0,563 | **-0,746** | -0,284 | 0,156 | 0,125 | -0,019 |
| DensityVariation | 0,484 | **-0,705** | -0,281 | -0,304 | -0,176 | 0,136 |
| EdfSurface [µm²] | **0,855** | 0,281 | -0,027 | -0,234 | -0,089 | -0,301 |
| EdfRoughness | **0,405** | -0,299 | -0,110 | -0,322 | -0,259 | -0,362 |
| Edf-Z [µm] | 0,281 | -0,359 | -0,020 | -0,073 | -0,258 | **-0,362** |
| *Values in bold correspond for each variable to the factor for which the squared cosine is the largest* | | | | | | |

| Ph | F1 | F2 | F3 | F4 | F5 | F6 | F7 |
| --- | --- | --- | --- | --- | --- | --- | --- |
| Area [µm²] | **0,949** | 0,289 | 0,046 | 0,102 | 0,002 | 0,022 | 0,014 |
| EqDiameter [µm] | **0,932** | 0,334 | 0,075 | 0,098 | 0,011 | 0,003 | 0,016 |
| VolumeEqSphere [µm³] | **0,940** | 0,249 | 0,019 | 0,102 | -0,004 | 0,040 | 0,012 |
| VolumeEqCylinder [µm³] | **0,927** | 0,259 | 0,021 | 0,102 | -0,016 | 0,006 | 0,004 |
| PerimeterContour [µm] | **0,946** | 0,290 | -0,130 | -0,032 | 0,032 | -0,030 | -0,009 |
| MeanChord [µm] | **0,844** | 0,363 | 0,302 | 0,230 | -0,018 | 0,030 | 0,063 |
| Length [µm] | **0,910** | 0,104 | -0,086 | 0,034 | 0,054 | 0,226 | 0,039 |
| Width [µm] | **0,740** | 0,494 | 0,214 | 0,127 | -0,036 | -0,239 | -0,015 |
| MaxFeret [µm] | **0,940** | 0,057 | -0,123 | 0,026 | 0,053 | 0,290 | 0,061 |
| MinFeret [µm] | **0,818** | 0,467 | 0,123 | 0,079 | -0,021 | -0,266 | -0,031 |
| Circularity | -0,375 | 0,087 | **0,780** | 0,456 | -0,102 | 0,081 | 0,137 |
| Elongation | 0,129 | -0,575 | -0,326 | -0,105 | 0,088 | **0,650** | 0,133 |
| ShapeFactor | -0,504 | 0,416 | **0,505** | 0,245 | -0,048 | -0,317 | -0,091 |
| Convexity | -0,405 | -0,082 | **0,538** | 0,360 | -0,017 | 0,276 | 0,016 |
| Roughness | -0,062 | -0,271 | **0,557** | 0,354 | -0,078 | 0,372 | 0,212 |
| MeanIntensity | 0,251 | **-0,928** | -0,070 | 0,214 | -0,105 | -0,085 | -0,078 |
| IntensityVariation | 0,378 | **-0,547** | 0,486 | -0,463 | -0,246 | 0,063 | -0,207 |
| MeanRed | 0,223 | **-0,872** | -0,075 | 0,308 | -0,095 | -0,019 | -0,283 |
| MeanGreen | 0,243 | **-0,921** | -0,066 | 0,184 | -0,122 | -0,113 | -0,001 |
| MeanBlue | 0,273 | **-0,938** | -0,064 | 0,136 | -0,089 | -0,115 | 0,055 |
| HueTypical | 0,089 | -0,299 | 0,060 | -0,294 | -0,085 | -0,291 | **0,569** |
| HueVariation | 0,224 | **-0,514** | 0,186 | -0,130 | 0,024 | -0,290 | 0,143 |
| MeanSaturation | -0,283 | **0,739** | 0,033 | 0,092 | 0,010 | 0,164 | -0,364 |
| MeanBrightness | 0,251 | **-0,928** | -0,070 | 0,214 | -0,105 | -0,085 | -0,078 |
| BrightVariation | 0,378 | **-0,547** | 0,486 | -0,463 | -0,246 | 0,063 | -0,208 |
| MeanDensity | -0,264 | **0,918** | 0,085 | -0,239 | 0,091 | 0,092 | 0,045 |
| DensityVariation | 0,241 | 0,019 | 0,530 | **-0,665** | -0,197 | 0,103 | -0,093 |
| EdfSurface [µm²] | 0,256 | -0,412 | 0,301 | -0,040 | **0,808** | -0,099 | -0,098 |
| EdfRoughness | 0,055 | -0,499 | 0,249 | -0,067 | **0,759** | -0,044 | -0,110 |
| Edf-Z [µm] | -0,038 | -0,226 | **0,331** | -0,215 | 0,259 | 0,090 | 0,225 |
| *Values in bold correspond for each variable to the factor for which the squared cosine is the largest* | | | | | | | |
